# Porous alloying-type particles for practical lithium-ion battery anodes

**DOI:** 10.1039/d5sc08594b

**Published:** 2026-01-26

**Authors:** Yiteng Luo, Sai Ho Pun, He Yan, Wei Liu

**Affiliations:** a Institute of New-Energy and Low-Carbon Technology (INELT), College of Carbon Neutrality Future Technology, Sichuan University Chengdu Sichuan 610065 China; b Department of Chemistry, The Hong Kong University of Science and Technology Clear Water Bay Kowloon Hong Kong 999077 China hyan@ust.hk; c State Key Laboratory of Intelligent Construction and Healthy Operation and Maintenance of Deep Underground Engineering, Sichuan University Chengdu Sichuan 610065 China weiliu@scu.edu.cn

## Abstract

Li-alloying-type anodes (Si, Sn, Ge, *etc.*) are potential candidates for high-energy lithium-ion batteries (LIBs), offering outstanding Li-storage capacity. However, their practical use is hampered by severe volume fluctuations during cycling, which lead to particle pulverization, an unstable interphase, and thus a shortened lifespan. Engineered porous structures have emerged as being key to solving these challenges. This review focuses on the porous alloying-type particles (ATPs) for LIB anodes. First, the structural evolution of ATPs with or without pores during lithiation is analysed using a graphite anode as a reference, highlighting the critical role of intraparticle rather than interparticle pores. Synthetic methodologies for fabricating porous ATPs are summarized and categorized into bottom-up, top-down, and transcription approaches, with special emphasis on their scalability for practical application. Recent progress in elucidating the in-cell evolution of pores and the key function of intraparticle pores is discussed in detail, emphasizing the contrasting effects of open *versus* closed pores. We also review representative diagnostic techniques for quantitative pore characterization, and the advanced binders or electrolytes that stabilize porous ATPs in the context of practical pouch or cylindrical cells. Lastly, we discuss cell-level considerations and operating procedures, outlining future research directions toward post-intercalation anodes for both liquid- and solid-state LIBs.

## Abstract 2

This review discusses porous alloying-type anodes for high-energy lithium-ion batteries, highlighting how engineered pores address expansion. It covers pore functionality, synthesis, characterization and their integration into practical cells.

## Graphical abstract

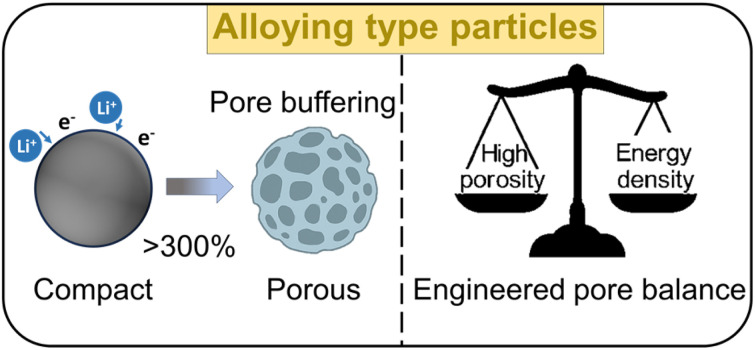

## 1. Introduction

Over recent decades, lithium-ion batteries (LIBs) have emerged as critical for integrating renewable energy into society, powering applications ranging from portable electronics to electric vehicles (EVs) and drones. To extend device duration and vehicle driving ranges within constrained weight and volume limits of batteries, the pursuit of higher energy density (Wh kg^−1^ and Wh L^−1^, respectively) has intensified research on next-generation electrode materials.^1–3^ While graphite particles remain the mainstream benchmark anode materials, their limited theoretical Li-storage capacity (372 mA h g^−1^) has prompted exploration of alternatives. Among these, alloying-type particles (ATPs)—including silicon (Si), tin (Sn), germanium (Ge), antimony (Sb), and aluminum (Al)—have garnered keen attention as promising alternatives to conventional graphite, offering tenfold higher capacity (up to >3580 mA h g^−1^) and at least 50% increase in cell energy-densities.^4^

Despite the advantages of high capacity, the widespread application of ATPs is heavily limited by their severe volumetric changes during the electrochemical lithium alloying/dealloying cycles. During the repeated cycling process, the colossal swelling exerts immense mechanical stress, leading to particle pulverization, loss of electrical contact,^5–7^ and continuous solid-electrolyte interphase (SEI) layer rupture and reformation that consumes lithium and electrolyte. This ultimately translates into rapid capacity fade and a much shortened cycle life. A community-wide consensus has been reached that mitigating this mechanical degradation is key for implementing ATPs in LIBs. Nano-sized ATPs have made considerable progress in mitigating particle cracking, *e.g.*, downsizing the Si to <150 nm is shown to lead to stable lithiation/de-lithiation cycles.^8–16^ However, the high specific surface area (SSA often >100 m^2^ g^−1^) and low compaction density (<0.2 g cm^−3^) of the nanoparticle-based electrodes contradict the criteria for practical LIB applications, setting aside the challenges of cost and scalability regarding nanoparticle synthesis. Introducing fine-tuned pores within micro-sized ATPs has emerged as a key alternative solution. It is shown to accommodate the volume changes and prevent destructive particle/electrode disintegration without compromising electrode SSA and compaction density.^17–20^

While porous ATPs are arousing intense research interest, fundamental understanding and optimal implementation of such a concept remains at a fairly early stage. A few crucial questions emerge: (1) how do the pores affect electrochemical alloying reactions? Although pores are supposed to dissipate the lithiation stress and accommodate expansion, higher porosity is not always guaranteed with lowered expansion and improved structural stability. The interaction of varying types of pores (porosity, pore sizes, pore volume, *etc.*) with the expanding primary particles, and their impacts on Li-diffusion and reaction kinetics, remain ambiguous. (2) What are the ideal synthetic means for pore engineering in ATPs? Although a number of synthetic strategies were developed and shown to offer unique advantages, the precise control over pore size and distribution, pore volume, and pore geometries remains rarely achieved in existing literature. (3) How to design pores in ATP-based electrodes for practical LIB cells: is more always better? In the context of practical LIBs, high-loading electrodes (>3 mA h cm^−2^) that are compact and thick represent the mainstream desire,^21–24^ and the electrode's porosity must suffice to contain expansion <20% throughout cycling.^14,25^ Achieving the optimal balance between high porosity and high compact density (and calendaring-compatibility) in high-loading electrodes imposes a critical challenge. We note that higher electrode porosity (“empty space”) results in not only a decay in cell volumetric energy density (Wh L^−1^),^16^ but also the excessive need for liquid electrolytes (g Ah^−1^) and hence compromises gravimetric energy density (Wh kg^−1^). These aspects require an in-depth rationalization of pore structures.

This review provides a comprehensive analysis of existing and emerging understanding of porous ATPs for LIBs. Existing reviews have focused on other aspects of ATPs. Jia *et al.*^26^ focused on the particle-interface and electrode hierarchy for improving the kinetic performance of the Si anode. Sun *et al.*^27^ reviewed the micron-silicon-based alloying anode for LIBs, while Lmtiaz *et al.*^28^ summarized recent progress in alloying-type anodes for potassium-ion batteries (PIBs). Very few articles have discussed the porous ATPs for LIBs, except for the topical review from Zhang *et al.* on the emerging significance of porous Si for Li storage applications.^29^ To date, the significance of pores in alloying-type anodes of LIBs is largely underappreciated. Bearing in mind the aforementioned questions, we here analyze the existing advances in constructing porous ATPs, and elucidate the corresponding structure–property relationships in the context of practical batteries. The trade-off between porosity and density is analyzed in detail while introducing advanced modeling simulations and *operando* analysis methods. The synthetic strategies, and advanced electrode auxiliary components and electrolytes for stabilizing ATPs were overviewed. Finally, future research directions to realize the full potential of ATPs were highlighted; as will be explained in detail, the judiciously engineered pores in ATPs can exert a major impact on both electrochemical reactions and battery performances.

## 2. Why do pores matter in ATPs?

**Fig. 1:**
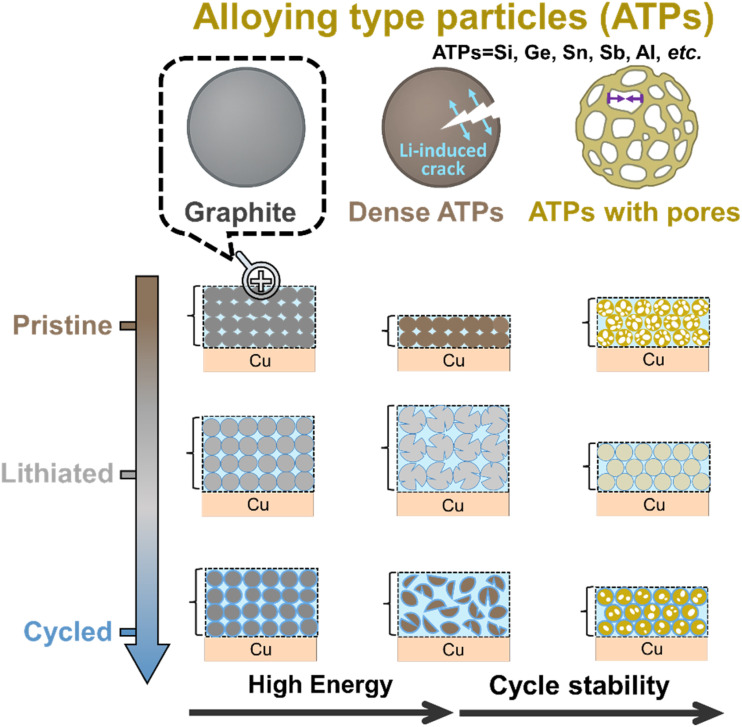
The comparison of graphite, dense ATP, and porous ATP electrodes. While dense ATPs reduce the electrode weight and thickness due to higher Li-storage capacity (mAh g^−1^, mAh cm^−3^), the associated expansion results in particle pulverization and electrode swelling. In contrast, ATPs with reasonable porosity offer emerging opportunities in ensuring long-term stability.

Since the early 1990s, when LIBs were first commercialized,^30^ graphite has been dominating the anode material market, due to its abundant sources, low production cost, and excellent cycling performance. While stability and low cost are well-suited for current LIBs, the limited theoretical capacity of graphite (372 mA h g^−1^) restricts cell energy density. And its operating voltage is very close to zero potential (0.05 V *vs.* Li^+^/Li), rendering it prone to lithium dendrite formation upon fast charging/overcharging, which leads to potential safety risks. Alloying type particles (ATPs, *e.g.*, Si, Sn, Ge, and Sb) store Li^+^ through reversible alloying reactions, enabling nearly an order of magnitude higher capacity than graphite (Si: 3579 mA h g^−1^,^31^ Ge: 1624 mA h g^−1^,^32^ Sn: 994 mA h g^−1^,^33^ Sb: 660 mA h g^−1^,^34^ and Al: 993 mA h g^−1^).^35^ This enables much lower loading levels (g m^−2^ or mg cm^−2^) and thickness of the electrodes (Fig. 1), resulting in much lighter and smaller cells (*i.e.*, higher gravimetric and volumetric energy density). Aside from that, ATPs can also offer suitable lithium insertion potential (0.4 V *vs.* Li^+^/Li for Si), and higher Li^+^ diffusivity (10^−7^ cm^2^ s^−1^ for Sn) that can translate to improved fast-charge capability and safety (avoiding Li-plating) compared to their graphite counterparts.

Despite their advantages, the major hurdle of applying ATPs is the severe volume expansion that originates from the insertion of lithium ions per host atom:• Si: Li + Si → Li_*x*_Si (*x* up to 3.75, forming Li_15_Si_4_), ∼280% volume expansion.^36^• Ge: Li + Ge → Li_*x*_Ge (*x* up to 4.4, forming Li_22_Ge_5_), ∼370% volume expansion.^32^• Sn: Li + Sn → Li_*x*_Sn (*x* up to 4.4, forming Li_22_Sn_5_), ∼260% volume expansion.^33^• Sb: Li + Sb → Li_*x*_Sb (*x* up to 3, forming Li_3_Sb), ∼147% volume expansion.^34^• Al: Li + Al → Li_*x*_Al (*x* up to 1, forming LiAl), ∼97% volume expansion.^37^

Severe volume expansion (97–370%) in ATP anodes during lithiation triggers three main failure modes: (1) particle mechanical degradation: repeated expansion/contraction causes particle pulverization, breaking electrical percolation networks. (2) An unstable SEI: an exposed cracking surface results in thick and inhomogeneous SEI growth, consuming Li^+^ and electrolyte. (3) Electrode disintegration: particle degradation and electrolyte swelling progressively mud-crack the active coating layer or detach it from current collectors, leading to a surge in impedance and the number of dead particles. These aspects collectively or more often interactively contribute to cell degradation.

ATPs with pre-engineered pores provide a promising solution to mitigate this issue. As shown in Fig. 1, comparing ATPs and porous ATPs, one may see that owing to the ingenious intraparticle pores in ATPs, the lithiation-induced expansion of ATPs can be much easier, as the outward growth of particle diameter is shifted to inward adaptive digestion. This paradigm improves the structural resilience of the particles and stabilizes the SEI, thereby extending the service life and dimensional stability of electrodes.

To achieve the abovementioned desirable functionality, particle pore engineering must be carried out that encompasses several key aspects:

(1) Porosity and pore volume: insufficient porosity inadequately accommodates Li-alloying-induced expansion, while excessive porosity compromises electrode volumetric compactness and calendaring compatibility. A careful balance is needed here, necessitating the precise control of porosity and pore volume in concert with the intrinsic expansion rates of the ATPs.

(2) Pore size: pores with diverging sizes offer distinct functions. For example, large-sized pores can allow electrolyte penetration, while mesopores and micropores (<2 nm) may not,^38^ and the meso-/micropores greatly impact Li-ion transport paths.^39,40^ To date, how the size of the pores affects SEI growth remains unclear, but surely tuning the pore size is key to controlling the mechanical and electrochemical response of ATPs to lithiation.

(3) Pore geometry and location: the uniform distribution of pores among the Li-alloying primary particles within ATPs is desirable, for it can achieve effective dissipation of lithiation expansion. In contrast, heterogeneously distributed pores may act as stress concentrators to initiate particle cracking. Moreover, the geometry of pores like openness/closeness to the electrolytes is arousing increasingly keen attention.^4,19^

The discussions delineated above outlined the pore-design principles for ATPs. Although the ideal pore structure for ATPs is still under debate, we attempt to highlight the core concepts of pore engineering in ATPs and to identify the key unresolved issues for realizing their adoption in practical LIBs. As extended cycle stability with reduced particle expansion represents a mainstream demand, ATPs with pre-planted pores have attracted increasingly widespread attention. However, how can such porous architectures be tailored and constructed in a scalable manner? In the next section, we will discuss the emerging synthetic methods of porous ATPs, with special emphasis on pore engineering.

## 3. Synthetic strategies for porous ATPs

**Fig. 2:**
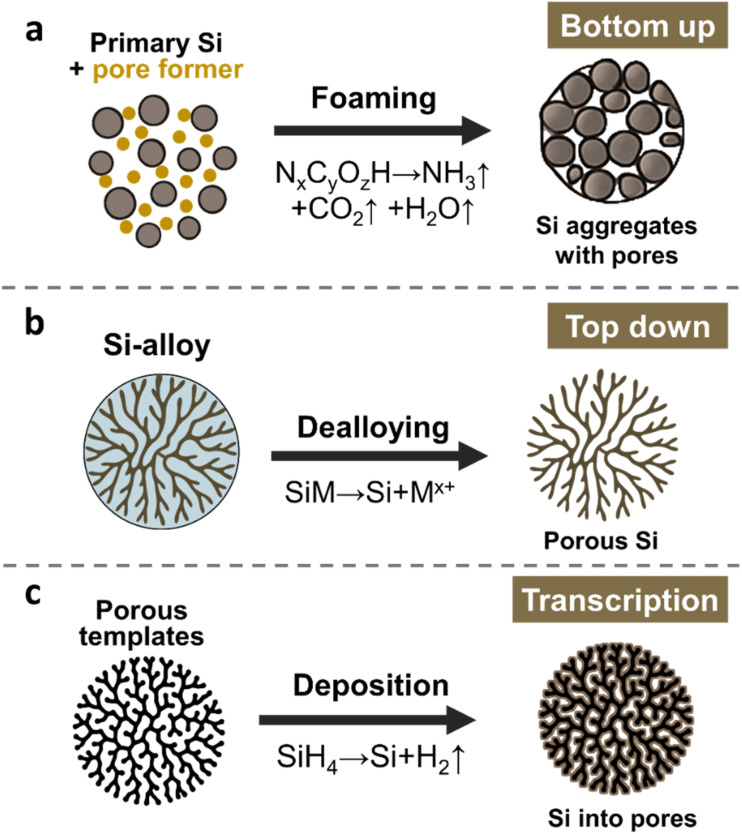
Primary synthesis strategy for constructing porous ATP anodes, taking silicon as an example: (a) bottom-up assembly of nano-Si with foaming agents, (b) top-down etching of Si-containing alloys, and (c) transcription of porous templates into porous Si.

Building on the recognition of pore functionality, diverse synthetic methodologies have been developed for porous ATP electrode materials. In the next section, we aim to review this progress and examine the corresponding mechanisms, advantages, and limitations. The synthesis methods of porous ATPs can be categorized into three, as illustrated in Fig. 2: bottom up, top down, and transcription. Each approach offers unique merits and demerits. Due to the intrinsic natural abundance and potential of lower cost, here we take Si as an example of demonstration and the main topic of discussion throughout the paper. However, most of the underlying scientific principles and technical routes apply to other ATPs as well.

The bottom-up strategy, illustrated in Fig. 2a, employs space-occupying agents (*e.g.*, polymeric compounds, nitrogen/ammonia-containing species, and soluble salts) to aggregate with Si primary particles and form secondary particles. These agents undergo controlled decomposition upon thermal pyrolysis (typically at 400–800 °C), or by adopting subsequent leaching, the agents were dissolved and removed, hence leaving porous architectures in the resulting secondary particles. Hence, these sacrificial agents are also often referred to as “pore formers”. Hence, one may deduce that the structural attributes of pores in the resulting ATPs can be controlled by adjusting the properties and content of pore formers, as well as the granulation and pyrolysis conditions. The nuanced trick lies in controlling the assembly structure of these agents and their homogeneous distribution in concert with the size and distribution of the primary Si. Managing the pyrolysis procedure and the residual carbons can also greatly impact the electrochemical performance.

The top-down approach (Fig. 2b) involves selective etching of a Si-containing alloy (*e.g.*, SiO_*x*_,^41,42^ FeSi,^43,44^ AlSi,^45–47^ and Mg_2_Si^48,49^), where the sacrificial components from the precursor alloys, often occupying 40–90 vol%, are removed, leaving a three-dimensional porous Si network. During this process, the porosity of the resulting Si framework is controlled by the volume fraction of the sacrificial components. The size, geometry, and location of pores originate from the metallographic structure of the Si-alloys. By selecting/controlling alloy composition, and metallographic and etching conditions, one may be able to obtain a porous ATP framework with differing pore structures. However, additional coating or compositing treatments are required to modify the etched framework to obtain final ATPs, as direct exposure of Si to liquid electrolyte is detrimental.^2,50–54^

The transcription strategy is depicted in Fig. 2c. This approach employs high-surface-area porous templates (often porous carbon), and then coating the templates with primary ATPs to deposit active Li-alloying species such as Si, Sn, or Ge. Physical vapor deposition [PVD],^55^ chemical vapor deposition [CVD],^56^ atomic layer deposition [ALD],^57^ and solution-phase depositions were often used. We note that the structural control of pores in the resulting ATPs relies on the mother templates, potentially providing unparalleled distribution uniformity of both pores and active primary ATPs. Prior study has shown that controlled nucleation from CVD can achieve silicon deposition uniformity down to a sub-nanometer level (∼1 nm), achieving a stable structure surviving prolonged cycling.^58^ Despite that, CVD-grown silicon–carbon anode materials have made their way into LIB applications in mobile electronics.^8,14,52,54,59,60^

When discussing the synthesis process of porous ATPs, special attention should be given to the quantitative characterization of the pore size, volume, geometry, and distribution. Powder-based tap density (TD), electrode-based compaction density (CD), and specific surface area (SSA) are indicators that are often used in industry practice, somewhat mirroring the porosity of the particles/electrodes. We note that until today, direct quantification of pores and their implications for electrochemical performances are not fully comprehended in existing literature. To bridge this gap, we provide representative porous Si–C ATP anodes and the reported electrochemical performances (compared and tabulated in Table 1), using silicon–carbon-based ATPs as typical cases.

**Table 1:** Typical performance of various Si–C based porous ATPs

Ref.	Synthesis methods	Specific capacity (mAh g^−1^)	ICE (%)	Areal capacity (mAh cm^−2^)	Rate-capacity retention@cycle	Tap density (TD, g cm^−3^)	Compaction density (CD, g cm^−3^)	SSA (g m^−2^)
61	Bottom-up	2500	82.6	2.7	100/67%	—	1	—
62		519	90.8	4.0	3.8 mA cm^−2^-95%@300	0.91	1.6	—
63		3389	83.9	4.4	1 A g^−1^-63%@800	—	2.0	—
64		1194	82.0	1.9	96%@200	0.86	1.2	3.3
65		2310	87.4	4.5	100/70%	4.15	1.75	—
66		1480	73.0	4.5	700/102%	0.53	—	44.3
67	Top-down	1271	80.3	4.5	1.2 mA cm^−2^-86%@100	0.80	—	—
68		537	91.4	3.5	0.5C-81.9%@200	—	1.6	5.85
69		3312	84.7	3.2	5 A g^−1^-86.3%@1000	—	—	112.4
70		530	∼81	2	0.5C-80%@450	0.48	1.6	8.8
71		916	64.0	0.9	0.5 A g^−1^-94%@200	—	—	165
70		2000	85	—	50/90%	0.93		8.8
		530	—	3.3	450/80%		1.6	
72	Transcription	844	84.0	3	0.75 mA cm^−2^-92%@500	0.5	1.4	61.5
73		∼700	87.8	2.8	0.6 A g^−1^-71%@400	—		—
74		1640	88.4	3.53	0.33C-95%@650	—	1.6	1678
75		731	90.9	3.6	0.5C-96%@50	—	1.6	1.42
75		732	90.9	3.6	50/96%	1.3	1.6	6.7

From Table 1, a clear negative correlation between high SSA and initial coulombic efficiency (ICE), as well as a clear positive correlation between compaction density and electrode areal capacity can be observed. Towards a genuinely well-performing ATP anode, where high ICE and high areal capacity (>2.5 mA h cm^−2^) are prerequisites, synthetic methodologies for achieving high porosity with little compromises in SSA and compaction density should be advocated. In the following section, the three categories of synthetic methods for porous ATPs will be analyzed in greater detail, pointing out the intercorrelation of pores with electrochemical behaviors in a case-by-case fashion. Subsequently, involvement of novel auxiliary components that help to stabilize the porous ATPs against deleterious structural degradation will be discussed as well, which include electrode binders, conductive agents, and electrolytes.

### 3.1 Bottom-up synthesis for porous ATPs

While nanosizing ATPs represents a major benefit in avoiding particle cracking, downsides of high SSA and low TD have drawn widespread attention.^8,76^ Challenges arise when attempting to use nano-sized ATPs in practical LIBs, which include intensified surface side reactions, lowered electrode porosity, and high electrolyte dosage. Assembling active nanoparticles into micro-sized secondary particles represents a mainstream advance in recent years. Based on the assembly medium (gas, liquid, or solid phase), the nano-to-micro synthesis approach can be further classified into three main sub-categories.

**Fig. 3:**
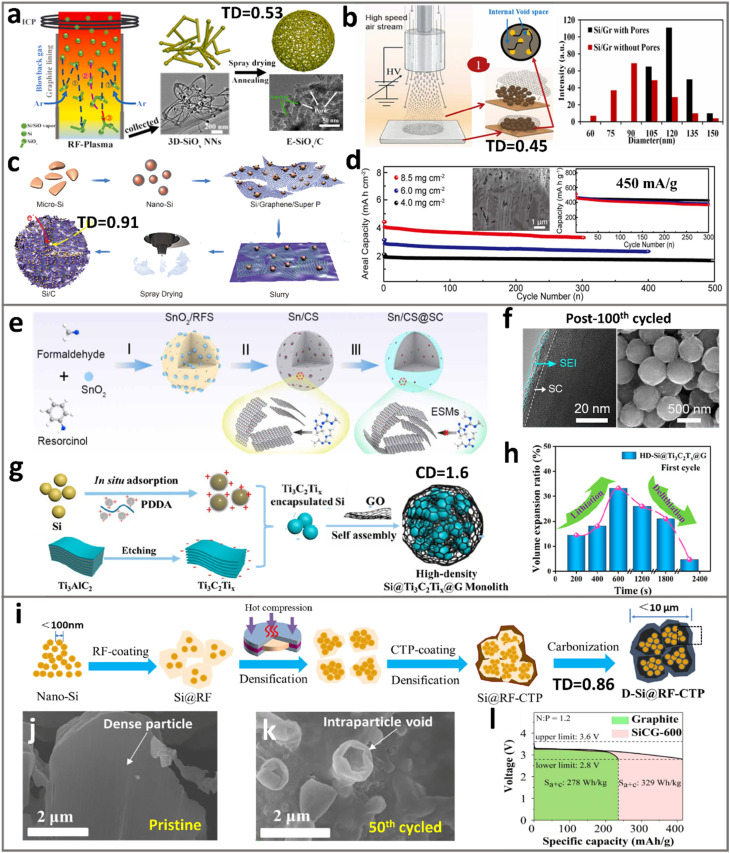
Bottom-up approach for porous ATP synthesis. (a) Schematics of SiO_*x*_/C micro-particles with pores *via* spray drying. Reproduced with permission from ref. 66. Copyright 2022 Elsevier. (b) Spray drying to prepare a Si/Gr/GNR hybrid by using polymers as a sacrificial pore former. Reproduced with permission from ref. 80. Copyright 2021 Wiley. (c) and (d) Spray drying to fabricate spherical C–Si particles, and its cycle performances at various mass loadings; the inset shows the cross-sectional SEM images of the porous particle. Reproduced with permission from ref. 62. Copyright 2018 Elsevier. (e) Porous Sn/CS@SC prepared *via* solution assembly and thermal reduction, (f) TEM and SEM images of cycled Sn/CS@SC. Reprinted with permission from ref. 82. Copyright 2025 Elsevier. (g) and (h) Schematic of preparing the high-density HD-Si@Ti_3_C_2_T_*x*_@G monolith *via* solvothermal self-assembly, demonstrating low lithiation expansion. Reprinted with permission from ref. 63. Copyright 2022 American Chemical Society. (i) Schematic of hot-pressing resorcinol–formaldehyde resin (RF) and coal tar pitch (CTP) to fabricate dense D-Si@RF–CTP particles, (j) and (k) SEM images of the pristine and cycled particles, highlighting the cycling-induced intraparticle pores, and (l) full-cell energy density by adding D-Si@RF–CTP into graphite. Reproduced with permission from ref. 64. Copyright 2023 Elsevier.

Spray drying is an effective gas-phase technique for granulating secondary particles, offering distinct advantages in high-speed output and cost-effectiveness.^77,78^ In the case of silicon–carbon composite particles, spray-drying is often implemented by co-dispersing SiNPs and carbon precursors into a liquid medium, followed by spray-drying such a slurry. During rapid solvent evaporation, the granulation of carbon precursors and embedded SiNPs occurs. These granules were further subject to pyrolysis, where the carbon precursor can be transformed into porous carbon, accompanied by the release of gases, which may originate from its molecular structure *per se* or the co-involvement of another pore former. Ouyang and Yuan *et al.*^66^ first prepared earbud-like SiO_*x*_ networks by using radio frequency plasma, and assembled SiO_*x*_ with sucrose into SiO_*x*_/C micro-particles *via* spray drying and carbonization (Fig. 3a). The pores are formed due to the interparticle “bridging” effect of SiO_*x*_ nanonetworks during spray drying. The ample pores in the formed secondary particles explain the excellent cycle stability (1480 mA h g^−1^ for 700 cycles at 2.0 A g^−1^). The SiO_*x*_/C micro-particles showed moderate tap density (0.53 g cm^−3^) compared to the true density of SiO_*x*_ and carbons (∼1.5–2 g cm^−3^), indicating the abundance of intraparticle pores.

It is noteworthy that the intraparticle pores and secondary particle structures are highly tunable with respect to the spray drying process, including the gas-flow rate, evaporation temperature, nozzle types, and pore former.^79^ Joshi *et al.*^80^ prepared Si/Gr/GNR hybrid anodes with a tap density of 0.45 g cm^−3^*via* air-controlled electrospray, where polyacrylic acid was used as a sacrificial pore-forming agent (Fig. 3b). The rich porous architecture surrounding the nanosized Si is thought to be responsible for improved capacity retention after 350 cycles. Yin and Guo *et al.*^62^ reported a spray drying method (>1 kg per batch) to assemble SiNPs into Si/C particles (Fig. 3c), showing considerable scalability that is relevant to analogous industrial particle production. The obtained spherical Si/C granules with 3D conducting networks have a compact structure with a high tap density of 0.91 g cm^−3^. Without the incorporation of a pore former, the intraparticle pores are formed purely from solvent evaporation. The Si/C particles delivered 3.2 mA h cm^−2^ after 300 cycles under high mass loading (8.5 mg cm^−2^) and high compaction density (1.6 g cm^−3^, Fig. 3d). A pouch cell adopting a 4 mA h cm^−2^ LiNi_0.5_Co_0.3_Mn_0.2_O_2_ cathode showed 87.5% capacity retention after 100 cycles. Here, the creation of nanosized primary particles is a key pre-step that often relies on sand or ball milling, with considerable simplicity and cost-effectiveness.^81^

While gas-phase synthesis facilitates scalable production of porous ATP materials, the properties of the produced materials are subject to the working gas flow. For systems involving nanosized Si, inner gas is required to avoid oxidation. This applies to nano-sized metals such as Sn and Ge as well. Liquid-phase assembly (*e.g.*, hydrothermal synthesis and sol–gel processes) offers superior isolation from air-oxidation or even tunability of pore structures *via* further reductive reactions. A representative example is demonstrated by Yu *et al.*,^82^ where an atomic Sn-incorporated sub-nanoporous hard carbon (Sn/CS@SC) is created for Li storage (Fig. 3e). The SnO_2_ colloids were first prepared *via* the modified Stöber method and served as self-sacrificial templates during carbonization, where subnanopores (0.4–0.8 nm) were formed during *in situ* reduction (SnO_2_ + C = nano-Sn + CO_2_). Hence, this achieves well-dispersed atomic Sn sites within the carbon matrix. Even after prolonged cycling, the particles retain intact spherical morphology, suggesting genuine stability of the porous particles (Fig. 3f).

Despite the advantages in controlling pore sizes, achieving uniform dispersion of nanoparticles in densely packed secondary particles remains a challenge. A refined approach utilizing electrostatic interactions can offer opportunities. Li *et al.*^63^ constructed densely packed Si-based particles by assembling MXenes and graphene layers with SiNPs, forming Si@Ti_3_C_2_T_*n*_@G hydrogels that was further subject to solvothermal treatment to create abundant mesopores (∼4 nm, Fig. 3g). As a result, the mesoporous network showed a SSA of 84.6 m^2^ g^−1^ and suppressed lithiation expansion from *in situ* TEM observation (Fig. 3h). Due to the absence of macropores, a high TD of 1.6 g cm^−3^ and high volumetric capacity (5206 mA h cm^−3^) were achieved. The electrostatic interaction between different components leads to capillary shrinking of the hydrogel during evaporation, key to achieving compact stacking and thus the densified secondary particles. In a separate study, Q. H. Yang *et al.*^61^ utilized the hydrogel “shrinking” mechanism to achieve tight assembly of Si microparticles (SiMPs) within graphene. The capillary shrinkage of a graphene hydrogel (∼20 × volume shrinkage) enables secondary particles with a CD of 1 g cm^−3^ to perform stably in full pouch cells under practical loading (3 mA h cm^−2^).

Solid-phase assembly offers opportunities for the tight packing of primary particles inside the secondary particles. Mechanical pressing represents an effective approach in this regard.^83^ Importantly, this densifying approach yields close-knit secondary particles that can survive harsh electrode calendaring. Liu and Chen *et al.*^64^ adopted hot-compression to assemble SiNPs into a dual-layered carbon matrix, yielding dense Si–C particles (D-Si@RF–CTP, Fig. 3i). A high temperature (220 °C) was adopted to first force the phenolic resin infiltrate into the interparticle space of SiNPs, where the subsequent cross-linking treatment at 270 °C (curing) solidifies the assembled compact structure. Then the cured pellets were crushed and milled into high TD primary particles, followed by the coating of pitch at an elevated temperature of 300 °C to form a compact exterior layer. The final Si–C particles were obtained after carbonization, exhibiting ∼10 µm diameter, high tap density (0.86 g cm^−3^), and low SSA (3.3 m^2^ g^−1^). Interestingly, due to the inherent porous nature of RF-derived carbon, cycle-induced pore formation was observed within such dense particles as shown in Fig. 3j and k. This unique pore generation mechanism allows harsh electrode calendaring to achieve high compaction density, delivering 20% energy density improvement of 2.5 mA h cm^−2^ LFP full cells by simply adding D-Si@RF–CTP into graphite (Fig. 3k).

The main superiority of the bottom-up strategy stems from the dimensional nano–micro synergy: while primary nano-particles shorten Li^+^ diffusion paths, the micrometre-scale secondary assemblies ensure high tap density and low specific area. The packing of nanoparticles into secondary particles is driven by a variety of processes: solvent evaporation in a spray, electrostatic interactions in a liquid medium, or external mechanical force in a solid. Not only did the assembly processes lead to diverse pore attributes, but it is also confronted with varying types of challenges for large-scale adoption. The gas-phase processes (*e.g.*, spray drying) hold the highest potential for scalability owing to the continuous operation and high throughput, but challenges remain in controlling the internal porosity and high cost of inert carrier gas and non-aqueous solvent (N_2_ and ethanol) to prevent oxidation of ATPs. Liquid-phase methods offer fine-tuned pores down to the (sub)nanometer scale but at mM concentrations, hence leading to the low yield and inherent difficulties in scaling-up. The solid-phase assembly method gives rise to highly-densified, calendaring-compatible secondary particles; nonetheless, it operates in an intermittent fashion and requires a liquid-based pretreat step, hence leading to a limited output and efficiency.

### 3.2 Top-down synthesis for porous ATPs

As bottom-up assembly made significant advances in fabricating porous ATPs, a shared feature of these approaches is the involvement of nanoparticles as building blocks. Nonetheless, taking Si as an example, the production of SiNPs usually hinges on a complex synthesis process, *i.e.*, prolonged sand milling of micron-sized Si or chemical pyrolysis of SH_4_. The limited manufacturing efficiency and high cost of nanoparticles posed severe challenges for large-scale applications. Alternatively, the top-down particle reconfiguration approach represents emerging opportunities to produce micron-sized Si-containing structures, exempt from the need for nanoparticle synthesis. This approach mainly involves (1) the extraction of porous Si from a micro-sized precursor; (2) coating or filling porous Si with other components, such as carbon. The size of the final particle should remain in the range of microns, while its porous Si backbone behaves analogously to conventional SiNPs.

Kim *et al.*^42^ adopted SiO_2_ as a precursor to prepare a porous Si framework *via* the magnesiothermic reduction-acid etching reaction. Then CVD-grown carbon was introduced into the porous structure, yielding Si/C microspheres. This “filled” structure led to a high TD of ∼0.8 g cm^−3^. Under *in situ* TEM examination, a volume expansion of ∼85% was identified in the Si/C microsphere, much lower than the theoretical ∼300% expansion for solid Si. Wang *et al.*^41^ first prepared silsesquioxane (SiO_1.5_) through a sol–gel process and then turned it into silicon and silica through thermal disproportionation reactions. After removing silica with a HF-etchant and then coating the carbon layers through CVD of acetylene, the micro-sized Si–C composite was fabricated. The resulting Si–C particles possess a volumetric capacity of 1088 mA h cm^−3^ with a tap density of 0.68 g cm^−3^.

**Fig. 4:**
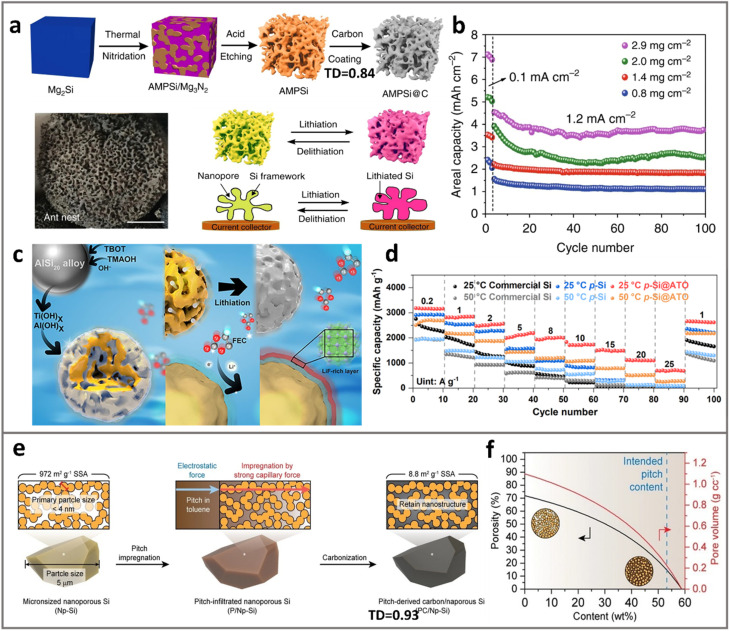
Top-down methods for porous ATP synthesis. (a) Schematics for the preparation of ant-nest-like micro-porous Si with carbon coatings (AMPSi@C), and (b) the cycle performances of AMPSi@C electrodes with various mass loadings. Reprinted with permission from ref. 67. Copyright 2019 Springer. (c) Schematics for porous Si with a dual alumina and titanium oxide layer (p-Si@ATO), whereas abundant pores were formed *via* etching off Al in the AlSi_20_ alloy. (d) Rate performance of p-Si@ATO. Reprinted with permission from ref. 69. Copyright 2025 Springer. (e) and (f) Schematics for the fabrication of pitch-derived carbon/nanoporous Si (PC/Np-Si), where pores were formed *via* etching the micrometer-sized disproportionate SiO, and the porosity/pore volume was further controlled by incorporating pitch in the pores. Reprinted with permission from ref. 70. Copyright 2021 Wiley.

One may note that creating porous Si from SiO_*x*_ involves magnesiothermic reduction and/or a HF-etchant, involving the dangerous toxic chemicals that impose serious challenges in scalability. Extracting porous Si from Si-containing alloys represents an alternative method if there is an intrinsic nano-sized metallographic structure of Si within.^85^ This top-down method utilizing varying types of Si-based alloys has aroused widespread attention.^43–49^ Removing the metallic components in these alloys can create porous Si and, in some cases, the residual metallic elements could enhance electric conductivity.^86^ As shown in Fig. 4a, Zhang and Huo *et al.*^67^ reported an ant-nest-like microscale porous Si (AMPSi) *via* thermal nitridation of the Mg–Si alloy in nitrogen (N_2_). Then, the removal of the Mg_3_N_2_ by-product in an acidic solution results in an AMPSi with a high tap density of 0.84 g cm^−3^ and a small SSA of 12.6 m^2^ g^−1^. After coating a 5–8 nm thick polydopamine-derived carbon layer, AMPSi@C delivered a capacity of 2843 mA h g^−1^ with an ICE of 80.3%. Under different areal mass loadings (0.8–2.9 mg cm^−2^), AMPSi@C could reach a high areal capacity of 7.1 mA h cm^−2^ at 0.1 mA cm^−2^ and retained 3.9 mA h cm^−2^ at 1.2 mA cm^−2^ after 100 cycles (Fig. 4b). The de-alloying process can be combined with controlled hydrolysis and dehydration to give fine-tuned porous architectures. As shown in Fig. 4c, Lv *et al.*^69^ developed a one-step synthesis of porous Si anodes by acid-etching the AlSi_20_ alloy. Hierarchical pores (4.9 nm mesopores and 50–80 nm macropores) were created, while an ultra-thin (∼2 nm) Al_2_O_3_–TiO_*x*_ (ATO) layer was also formed through hydrolysis. The ATO layer promotes a LiF-rich SEI that enables ultrafast Li^+^ transport and exceptional rate capability (Fig. 4d, 692 mA h g^−1^ at 25 A g^−1^).

Yi and Zhang *et al*.^70^ reported a pitch impregnation method to fabricate a Si/C composite (termed PC/Np-Si, as shown in Fig. 4e). Porous Si was obtained first by etching off the Si–O components of a SiO_*x*_ microparticle, using HF as the etchant. After pitch-impregnation and carbonization, the micron-sized PC/Np-Si particles showed a low SSA of 8.8 m^2^ g^−1^ and high TD of 0.93 g cm^−3^, while the nano-porous Si precursor (Np-Si) showed 972 m^2^ g^−1^ and 0.48 g cm^−3^. The pitch-toluene solution was forced to infiltrate into the pores of Si under vacuum. The porosity and pore volume in the composite particles can be well tuned by controlling the pitch content up to 53 wt% (Fig. 4f). Hence, the resulting PC/Np-Si particles depict 60.6% expansion upon full lithiation without showing fractures. The mitigated expansion mainly arises from porous structures of primary Si, leading to 80% capacity retention after 450 cycles in full cells with NMC cathodes (2 mA h cm^−2^).

One may note that the aforementioned studies all involve a carbon coating treatment on porous Si, being a critical step to prevent the sintering of Si nanostructures, as thermal- or electrochemical-induced sintering of Si occurs during pyrolysis or battery cycling. The carbon coating is also critical to improve electrode–electrolyte interfacial stability, as direct exposure of Si to the electrolyte is highly deleterious. However, we note in many cases that the improper carbon coating can result in low TD and high SSA that jeopardize battery performances. For instance, in the work shown in Fig. 4e and f, control groups of Si–C composite particles obtained by filling acetylene-derived carbon on porous Si result in a lower ICE (76%) and lower TD (0.68 g cm^−3^),^41^ much inferior as compared to those of PC/Np-Si^70^ (ICE = ∼80%, TD = 0.93 g cm^−3^). Perhaps the most prominent benefit of the top-down synthesis approach, as compared to the bottom-up approaches, is that it waives the need to fabricate nanosized ATP primary particles. Nonetheless, caution needs to be exercised due to the use of specific etchants, *e.g.*, HF, where serious concerns about the cost and hazardousness can be major impediments for industrial adoption.

Top-down etching methods enable a uniform distribution of ATPs and pores, and they directly circumvent the need for arduous synthesis of nanoparticles that is often expensive and has low throughput. The pores were formed *via* etching off the non-Si components (*e.g.*, SiO_2_, Mg_2_Si, and Al) out of the alloy; hence the pore structure (size, geometry, *etc.*) is mostly “inherited” from the metallographic morphology of the Si-alloy. This allows the precise pre-design and control of pore architectures at the early stage of precursor synthesis. However, this path presents its own formidable scalability and safety challenges, and heavily relies on erosive chemicals involved in magnesio-thermic reduction and acid etchants. This raises concerns regarding safety, the cost of waste treatment, and operational danger. To achieve balanced performance, cost, and scalability, the viability of top-down synthesis hinges on the development of greener and safer Si-alloys-etchant pairs, as well as novel reaction routes and reactors.

### 3.3 Transcription synthesis for porous ATPs

Unlike bottom-up or top-down synthesis methods, transcription-based synthesis circumvents the need for the etching process and the difficulties in making nanoparticles. This approach in general involves a two-stage process: (1) the configuration of porous structures in micron-sized particles for use as templates, and (2) chemical deposition of active ATPs into the pores, “transcribing” the structural attributes of templating, including pore size, geometry, distribution, *etc.*

**Fig. 5:**
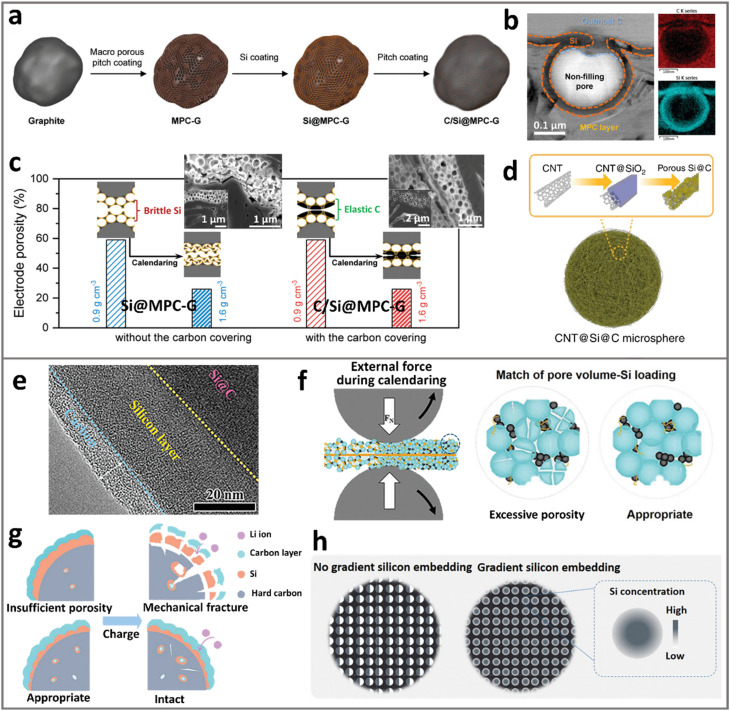
Transcription approach to fabricate porous ATPs. (a) Schematic and (b) HRTEM images of a Si-coated macroporous carbon–graphite composite (C/Si@MPC–G); (c) calendaring compatibility of C/Si@MPC–G w/wo carbon coating. Reprinted with permission from ref. 75. Copyright 2020 Wiley. (d) Schematic for the synthesis of CNT@Si@C microspheres, through emulsion-based assembly, aluminothermic reduction, and CVD carbon coating, where the pores originate from the stacking spaces between CNTs. Reprinted with permission from ref. 72. Copyright 2020 Springer. (e) The TEM image of the high-Si-loading Si/C composite, fabricated through step-by-step deposition of silane and C_2_H_2_ into a porous hard carbon matrix. (f) and (g) Appropriate porosity design is key for ATP anodes as excessive porosity results in poor structural stability against calendaring, while insufficient porosity leads to poor structural stability upon lithiation. Reprinted with permission from ref. 74. Copyright 2024 Wiley. (h) Gradient Si distribution into carbon pores, offering further opportunities in harmonizing the pore volume with Si loadings. Reprinted with permission from ref. 87. Copyright 2025 Royal Society of Chemistry.

Graphite exhibits excellent electric and electrochemical performances, serving as an ideal template for accommodating ATPs. Cho *et al.*^75^ created macropores on graphite and then filled the pores with Si (first) and carbon (second) layers *via* CVD, forming an architecture that consists of a macro-porous graphite interior, ultrathin Si interlayer, and outermost carbon covering (Fig. 5a and b). The obtained silicon–carbon composite (termed C/Si@MPC–G) showed impressive calendaring compatibility; the elastic carbon covering is found to be key to retain its intraparticle macropores even after harsh electrode calendaring (Fig. 5c). As a result, C/Si@MPC–G exhibited an ICE of 90.9% and capacity retention of 95.6% after 50 cycles under practical conditions (high CD of 1.6 g cm^−3^, 3.6 mA h cm^−2^). Furthermore, a full-cell with NCM622 achieves energy densities of 333 Wh kg^−1^ and 932 Wh L^−1^.

As compared to graphite as a mother material, carbon nanotubes or carbon fibers offer inherent structural design flexibility as transcription templates for pore engineering. Zhang *et al.*^72^ developed “yarn-ball-like” porous CNT@Si@C microspheres through a multistep process: sol–gel emulsion coating of SiO_2_ on CNTs to form encapsulated coaxial cables, followed by aluminothermic reduction of SiO_2_ to Si and secondary carbon-coating *via* CVD. The microspheres endow improved mechanical strength (>200 MPa) thanks to the high strength of CNTs, preserving spherical morphology and 1.2 g cm^−3^ tap density. This engineered structure features optimized porosity (20 nm in average) and minimal lithiation swelling (<20% at 100% SOC). Stable cycling at practical loadings (3 mA h cm^−2^, ∼750 mA h g^−1^, 92% retention after 500 cycles) in half cells was achieved.

The in-pore deposition approach on CNT/CNF-based architectures allows the introduction of ample pore structures; it inherently requires an assembly step to obtain micron-sized particles. Alternatively, direct Si deposition onto the porous carbon micro-framework has been explored, where the pore structures of the porous ATPs depend on the porosity of the mother framework and deposition procedure. Du *et al.*^74^ sequentially deposited Si and C onto porous hard carbon templates with varying pore volumes to synthesize composite Si–C secondary particles (Fig. 5e). The results demonstrate that the porosity of the porous hard carbon critically determines the mechanical properties and structural stability of the resulting composite particles. Specifically, larger pore volumes (HC-1) showed excessive porosity, leading to pore collapse under calendaring stress and capacity fading (Fig. 5f), while smaller pore volumes (HC-0.6) provided insufficient space for the in-pore growth of silicon, resulting in out-of-pore Si growth that is deleterious for electrochemical stability (Fig. 5g). The hard carbon with optimal porosity (HC-0.8) can effectively balance these competing factors, allowing sufficient in-pore growth of Si without compromising particle integrity to achieve cycling stability up to 650 cycles. These findings outline the critical role of porosity in the templates.

The in-pore deposition processes are often coupled with a carbon coating step. A step-by-step deposition of the active ATP materials and carbon coatings may offer further opportunities. For C–Si composite fabrication, one may utilize mixed precursor streams during pyrolysis, *i.e.*, silicon sources (SiH_4_, ethylsilane, or silicon chlorides) and carbon sources (acetylene, methane, or ethylene) mixtures. Such concurrent deposition with intensified flow and deposition rates could potentially enhance production output. Recently, commercial practices have increasingly advocated gradient nano-silicon embeddings by introducing dynamically modulated gas streams (*e.g.*, from Group 14, as shown in Fig. 5h). This controlled deposition results in a gradient structure with a silicon-rich core that gradually transits to a carbon-rich outer surrounding within the pores. Such a Si–C gradient is designed to redistribute lithiation stresses and improve structural durability upon cycling. Here, we note that the production rate of the CVD-based transcription method is often limited to low production output rates due to the intrinsic low molarity (mol L^−1^) of the gas reactant. Moreover, the thermal stability of the produced Si–C composites is limited as a nanosized amorphous Si layer can react with carbon to give SiC at >800 °C, while conventional crystalline nano-Si remains stable at ∼1200 °C. Given this disparity in intrinsic thermal stability, the carbon coating requires careful selection, including precursors and pyrolysis temperature.

Transcription synthesis presents a highly controllable method that offers exceptional potential for precise spatial control over nano-sized primary ATPs into pores with diverse geometry, size, and distribution. Achieving an optimal template requires a careful balance between sufficient pore accessibility and volume (*e.g.*, for CVD-based loading), and mechanical and electrical properties of pore walls. The deposition of primary ATPs within template pores is governed by the mass transport in pores and pyrolysis kinetics in confined spaces, which clearly require more fundamental research endeavours. However, great challenges remain for their further scale-up. That includes the high equipment costs, low production throughput (due to the low molarity/density of gaseous precursors), and dangers/hazards of gaseous precursors (SiH_4_, C_2_H_2_, *etc.*). Future advancement in this direction hinges on the development of advanced deposition equipment (*e.g.*, pulsed or plasma-enhanced CVD and fluidized bed), as well as exploiting potential alternatives, safer and more cost-effective than SiH_4_ and porous carbon.

### 3.4 Beyond-Si porous ATPs

While the aforementioned synthesis strategies mainly focus on Si-based porous ATPs, their successful translation across different alloying-species necessitates careful evaluation. Although diverse ATP systems vary in chemical, electrochemical, and physical characteristics, the core principles of creating porous ATPs, including bottom-up assembly, top-down and transcription synthesis, remain universally applicable. However, system-specific adaptations are often required.

Table 2 summarizes the properties of each type of ATP, highlighting their feasibility for constructing porous architectures *via* varying synthetic pathways. A few key aspects we consider key to transferring the abovementioned synthetic concepts and methodologies into beyond-Si systems (*e.g.*, Ge, Sn, Sb, and Al) are listed below, using graphite as a benchmark:

**Table 2:** Material properties for porous ATP (Si, Ge, Sn, Sb, and Al) synthesis

Materials	Expansion rate (%, upon full lithiation)	Capacity (mAh g^−1^, mAh cm^−3^)	Electrical conductivity (S cm^−1^)	Synthesis pathway adaptability		
				Nanoparticle synthesis	Etching selectivity	Chemical deposition
Si	∼280	3579, 8340	∼10^−6^	Medium	Acid	Mature SiH_4_, SiCl_4_
Ge	∼370	1624, 8636	∼10^−2^	Difficult	Amphoteric	Costly GeH_4_
Sn	∼260	994, 7209	∼10^4^	Facile	Facile	Unstable SnCl_4_, SnH_4_
Sb	∼147	660, 4409	∼10^4^	Medium	Facile	Unstable SbH_3_
Al	∼97	993, 2681	∼10^5^	Difficult	N/A	N/A
Graphite	∼10%	372, 841	10^2^–10^3^	N/A	N/A	N/A

(1) Electrochemical properties. This includes the expansion rate upon full lithiation and the gravimetric and volumetric capacity. The volume expansion rate (from ∼97% of Al to ∼370% of Ge) imposes a material-dependent constraint on pore design. For high-expansion and high-capacity materials (*e.g.*, Si and Ge), creating compliant pores (>50% porosity) is essential to digest the lithiation strain. However, this design directly compromises volumetric energy density. For example, Ge offers high volumetric capacity (8636 mA h cm^−3^) partly due to its high density, yet its extreme expansion (370%) necessitates a very high porosity (*e.g.*, 370/470 = 78%). In contrast, for lower expansion materials like Al, porosity may mainly serve as electrolyte infiltration tunnels, but not as a stress buffer, requiring a different pore size.

(2) Electrical conductivity. Conductivity varies by orders of magnitude across differing ATP systems, spanning from ∼10^−6^ to 10^5^ S cm^−1^. This fundamentally determines the selection of synthesis paths. For semi-conducting Si and Ge, the porous ATPs have to be integrated with a conductive matrix to ensure adequate electron transport, such as carbons. Conversely, this is not necessary for high-conductivity metallic Sn, Sb, and Al; however, delicate surface treatment is needed to ensure the electron pathway throughout cycling and isolate potential interfacial side reactions.

(3) Synthesis pathway adaptability. The adaptability of synthesis routes for varying ATP systems is strongly material-dependent. Here we attempt to analyse their corresponding adaptability in bottom-up, top-down or transcription-based methods:

(a) Ease of nanoparticle synthesis. Synthesis of nano-Si is very well established, including CVD-deposition from SiH_4_ and high-energy milling, yielding nano-Si with varying particle sizes. However, the nanonization for Sn, Ge and Al is highly challenging from milling due to ease of oxidation and ductility. It is facile for making Sn and Ge from CVD deposition of SnH_4_ and GeH_4_, but with extremely high cost.^88,89^ Ge and Sn nanoparticles produced in this way can cost >1000 $ per kg, making it unsuitable for battery application. The production of CVD-grown Al is very difficult, partly due to the fact that AlH_3_ is a solid.^90^

(b) Etching selectivity from alloys depends primarily on the differing reactivity between the desired ATP and the sacrificial alloying component. It is favorable for Si and Sb, where acid-based etching can selectively remove sacrificial metallic-phases (*e.g.*, Mg and Al) with higher electronegativity. However, Sn, Ge, and Al exhibit poor selectivity due to their reactivity with both acids and alkali, making this route challenging.

(c) Chemical deposition is a key prerequisite for the transcription method and relies on a gaseous precursor. This is frequently adopted for depositing Si from SiH_4_, SiH_2_Cl_2_, SiHCl_3_ and SiCl_4_.^87,91–93^ The chemical deposition of Ge, Sn, and Sb in a porous substrate is rarely explored by far, partially due to the lack of proper precursors, and the CVD processes are either high cost or dangerous (GeH_4_, SnH_4_, and SbH_3_).

## 4. Modelling and characterization of ATPs

The synthetic strategies discussed above provide the means to deliver porous architectures across varying ATP systems. The ultimate challenge, however, lies in translating these theoretical advantages into real-world cell performances. This necessitates electrode engineering as well as modelling/diagnostic tools to capture the in-service function of ATPs. While the preceding sections have detailed the synthesis of porous ATPs, we acknowledge the fundamental trade-offs in pore design. Although intentionally introduced intraparticle pores can mitigate the inherent volume expansion of Li-alloying reactions, excessive pore formation introduces a number of drawbacks. These include mechanical instability that can cause particle fracture and collapse against calendaring or cycling. More importantly, electrode porosity compromises cell volumetric energy density. Hence, the judicious design and control of pores in ATPs becomes critical in practical high-energy LIBs, as electrodes are getting progressively thicker and more compact. Volumetric capacity (mAh cm^−3^) or energy density (Wh L^−1^) is determined by dividing the capacity/energy by the volume of the electrode/cell. While adding high-capacity ATP materials (*e.g.*, silicon) into graphite anodes increases the numerator (capacity), excessive porosity inflates the denominator (volume). Beyond a certain threshold, this trade-off yields negative returns, where the volume increase outweighs capacity gains, ultimately reducing volumetric energy density.

**Fig. 6:**
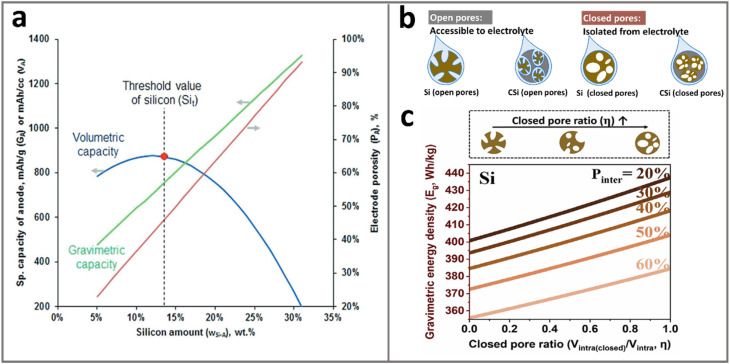
(a) Trade-offs between gravimetric/volumetric capacities under varying silicon contents and electrode porosity, revealing the optimal value (dashed contours) located at a point where a further increase in Si contents is accompanied by an increase in porosity. Reprinted with permission from ref. 14. Copyright 2019 Wiley. (b) Pore architecture that determines the pore–electrolyte relationship: particles with open pores are accessible to liquid electrolyte while closed pores are not; (c) transforming open pores to closed pores leads to higher cell energy densities due to the reduced electrolyte dosage. Reprinted with permission from ref. 94. Copyright 2024 Wiley.

For example, a theoretical model for graphite/Si anodes has shown that under practical 5% swelling constraints, as shown in Fig. 6a, the volumetric capacity of the anode follows a parabolic trend with silicon content reaching a peak at 11.68 wt% Si before declining due to the concomitant porosity increase.^14^ This pattern underscores the importance of reasonable pore engineering as well as the highest achievable energy density of ATP-based LIBs, which can be achieved at an anode capacity of ∼800 mA h g^−1^. Gravimetric energy density (Wh kg^−1^) is influenced by the electrolyte in the pores. Since the electrolyte accounts for a significant proportion of the weight of the battery, porous ATPs require a higher electrolyte dosage (g Ah^−1^) to ensure sufficient infiltration and ionic conduction. This is especially the case when solid electrolytes are employed due to their high density: >2 g cm^−3^ for sulfides and >4 g cm^−3^ for oxides as compared to liquid or polymer electrolytes (∼1.2 g cm^−3^). The increase in electrolyte dosage can significantly compromise the gravimetric performance. In this regard, the need for pore quality outweighs pore quantity. This issue was thoroughly investigated by Koratkar and Liu *et al.*,^94^ who defined pore quality based on its openness or closeness, *i.e.*, its accessibility or isolation to electrolytes. As shown in Fig. 6b, one can see that increasing the closed pore ratio, *i.e.*, more and more pores become “closed” and inaccessible to electrolytes, leads to an eye-catching increase in energy density due to the reduced electrolyte dosage. In this way, the gain in energy density is even more profound than the increase in Si contents *per se*. The pore engineering is projected to enable >400 Wh kg^−1^ cells with 2 g Ah^−1^ electrolyte dosage with 15 wt% Si-based LIBs (Fig. 6c).

### 4.1 Modeling tools for porous ATPs

Modeling and simulation of porous ATPs usually involves structural factors, spanning from the size, distribution, geometry, and stoichiometry of both the pores and the chemical composition. The electrochemical-mechanical interplay serves as a core concept that dictates the function and evolution of pore architectures. There are well-established fundamental underpinnings to construct models for varying purposes, such as the Butler–Volmer equation to describe the polarization and reaction kinetics,^95,96^ Fick's laws to depict the bulk-phase and solution Li-concentration/diffusion,^97^ and or mechanical properties of Li-alloys.^95,98,99^ Constraints and boundary conditions including the pore size, pore wall geometry and volume expansion ratio are usually assumed to make the calculation solvable. A representative approach is made by Zhang *et al.*^100^,who applied continuum media mechanical calculations in a three-dimensional mesoporous silicon sponge (MSS) that consists of thin Si walls and large pores. Assuming stress-free and volume conservation, they found that if the MSS particles have an initial pore radius of >10 nm and porosity of >80%, the lithiation-induced volume change of the MSS microparticle can be reduced to 0. Although the detailed computational framework is beyond the scope of the current discussion, such electrochemical-mechanical interplay highlights the key structural attributes of porous ATPs in the range of nanometers to millimeters.

Density functional theory (DFT) and molecular dynamics (MD) focus on much smaller dimensions down to the atomic scale. DFT simulation yields the energy-related derivatives of a system based on its electronic structures, and MD, by solving Newton's equation of motion, can picture the dynamic phase space. Adopting the DFT method, early studies identified a series of changes in mechanical properties of Si during cell operation, much of which is summarized as lithiation-induced softening.^101,102^ Later on studies further combine DFT with MD to capture the actual Li-ion transport process and correlate that with plastic deformation, agreeing with the *in situ* measured stress evolution in thin Si-films.^103,104^ However, MD or DFT based simulations are often limited to systems with tens to hundreds of atoms. Benefiting from the fast development of new algorithms (or artificial intelligence) and high-performance supercomputers, the scope of these methods can now be expanded to the nanoscale.

Understanding the mechanical response of porous ATPs is challenging considering the relatively large size of pores (often in microns), and the varied properties of Li-alloys with different Li-concentrations, *i.e.*, different states of charge (SOC), further complicate this issue. Taking Si as an example, the lithiation of crystalline Si is known to be anisotropic,^105–107^*i.e.*, the Li-diffusion and reaction phase boundary will preferentially propagate along the zone axis of 〈110〉.^95,107,108^ The anisotropic deformation, non-linear strain–stress response, and stress concentration in local areas are the main driving forces to initiate particle cracking and hence the collapse of the physical model.^109^ In contrast, the finite element method (FEM) considers a deformable body within 3-dimensional space, where the position of a typical particle (element) is defined by means of a vector. An infinite-dimensional system encompasses the position of all of its particle (element) points, offering unique advantages in integrating all small regions into a larger system.^97,110^ A dimensional reduction of the target system can be achieved by placing one or multiple restrictions on the admissible motions of the body. In some cases, the lithiation-induced expansion of Si was modeled in a way mimicking the thermal expansion using a J2-flow theory, to further simplify the calculation.^111^ Here for conciseness, we advise readers to turn to previous FEM-focused reviews.^111,112^

**Fig. 7:**
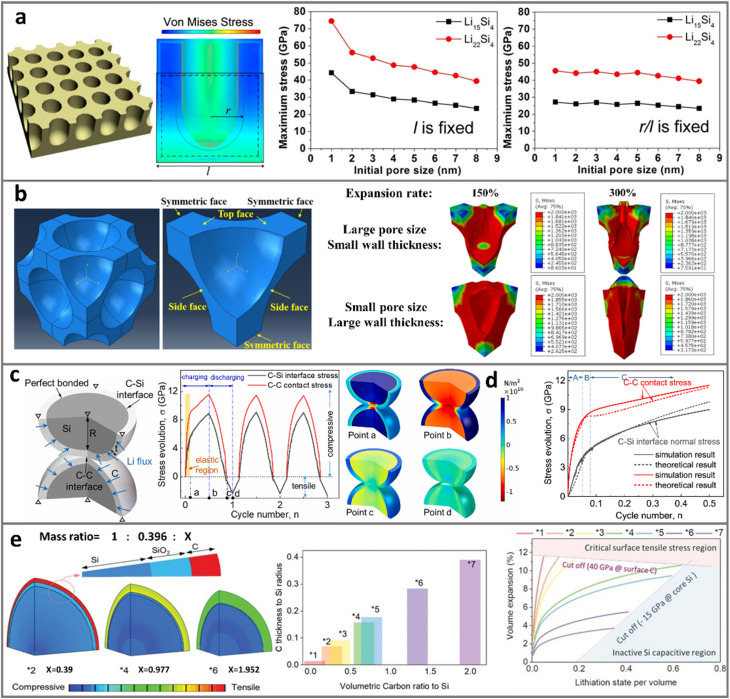
Finite element simulations of porous ATP anodes. (a) The maximum lithiation stress in porous Si correlates with the pore size and pore-to-pore distance. Reprinted with permission from ref. 113. Copyright 2012 American Chemical Society. (b) Stress distribution of Si with spherical pores, highlighting the critical role of pore size and wall thickness. Reprinted with permission from ref. 99. Copyright 2020 American Chemical Society. (c) and (d) Interfacial stress evolution at the two neighboring Si–C particles with core–shell structures, where stress at the interparticle C–C interface is much larger than that at the intraparticle C–Si interface. Reprinted with permission from ref. 118. Copyright 2019 Elsevier. (e) For Si-particles with SiO_2_ and C dual-shells, the maximum lithiation capacity is determined by the balanced tensile stress on the shell and compressive stress on the Si core. Reprinted with permission from ref. 119. Copyright 2020 Wiley.

Zhou *et al.*^113^ modeled lithiation-induced strain of porous Si *via* FEM by simplifying the pore network into an idealized, periodic array of cylindrical pores. Fig. 7a (left) shows a representative unit with a specified pore size and pore/edge ratio. By applying specific boundary conditions and assuming isotropic elastic expansion behavior, this work demonstrates a one-way stress evolution driven by lithium diffusion (*i.e.*, diffusion induces strain, but stress feedback on Li-transport is omitted). It is found that when the pore-to-pore distance (*l*) is fixed, reducing the pore size from 8 nm to 1 nm will lead to greatly aggravated stress levels and cause mechanical fracture. However, by defining a parameter as the ratio of the pore radius *versus* the pore-to-pore distance (*r*/*l*), and fixing the *r*/*l* ratio at 1/3, the maximum stress is almost invariant to the pore size (Fig. 7a right). As high porosity and large pore size are, in general, expected to stabilize Si, this work underlines that the spacing of pores also matters. Compared to sparsely distributed pores, “crowded” pores are more effective in stress dissipation.

Xia *et al.*^99^ chose 1/8 of the 3D porous Si as a representative volume element (RVE, Fig. 7b left) for FEM modeling. In their simulation, they modeled the complex chemo-mechanical coupling *via* a thermo-mechanical analogy, where lithiation-induced expansion is mimicked by thermal expansion. A boundary condition is adopted, where the side face along the vertical direction is fixed and the top surface is free for expansion. By reasonably mimicking the mechanical response of a periodic porous architecture, it is shown that macropores (300–400 nm) play a critical role in stress dissipation and structure stabilization of the lithiating silicon. In contrast, a structure with smaller pores not only exhibits higher stress levels at the same expansion rate, but also causes severe deformation of Si (Fig. 7b right). It is shown that overall, thin Si walls with a large pore size are more preferable. This agrees with a recent study where both simulations and experiments on porous Si with 15 different pore/wall ratios were studied.^114^ It is shown that the pore/wall ratio with 0.5 (Si wall of 600–700 nm) is “ideal”, effectively accommodating the expanding Si.

Adopting porous Si structures as a model system for FEM simulation points to the critical role of size, volume, geometry, and distribution of pores. However, as direct adoption of high-SSA (>10 m^2^ g^−1^) porous Si is impractical, carbon–silicon composite particles represent a more practical choice.^115^ Analysis of stress distribution and structural evolution of the intraparticle and interparticle C–Si interface is of great interest. In practical electrodes with densely packed C–Si particles, the simulation of particle-to-particle collision and movement is highly useful.^116,117^ Xu *et al.*^118^ conducted axisymmetric FEM on the silicon–carbon (Si–C) core–shell particles (shown in Fig. 7c left). Several key assumptions and boundary conditions were adopted: (1) the Si–C interface is assumed to be perfectly bonded without interfacial debonding or sliding. (2) The carbon shell is purely elastic and free of fracture. (3) Strain-rate-dependent viscoplasticity is considered for the Si core, but rate effects on the shell and interface are the same. These setups enable a tractable analysis of stress evolution and particle interaction, but excluding the degradation due to shell cracking or fatigue. As a result, it found that when two core–shell Si–C particles are in close contact, each of which possesses a 10 nm thick C shell, the stress at the C–C contact will be much higher than that at the Si–C interface (shown in Fig. 7c middle and right). This indicates that the C–Si particle disintegration starts at the C–C interface and propagates inward, rather than outward cracking originating from the C–Si core. As the interparticle contacts are inevitable in calendared electrodes, this work suggests that directing expansion inward is effective to prevent Si–C particle cracking. This work also reveals that thicker carbon shells, ones that are less likely to fracture, would help reduce the permanent structural damage of C–Si particles.

Nonetheless, excessive thickening of the carbon coating layer hinders Li diffusion of C–Si particles.^120^ S. Y. Kim and J. Cho^119^ adopted FEM to simulate a C–Si particle model with 10 nm-SiO_2_ and carbon layer double shells (Fig. 7e). They also adopt a thermal-mechanical analogy, and assume linear elastic material behavior for all phases (Si, SiO_2_, and C). To confine the deformation within the elastic regime, they introduced a stress-limited lithiation criterion, whereby Li-insertion ceases once the tensile stress in the outer carbon layer reaches a predefined elastic limit (∼40 GPa). This approach provides valuable guidelines to identify an optimal carbon content for mechanical stability. In their results, increasing the C thicknesses from *1 to *7 with respect to the Si radius, will result in Si : C ratios from 1 : 0.073 to 1 : 2.949. It is found that the deficient C layer (*2) could generate extremely strong tensile hoop stress and cause particle fracture. While with an excessively thick C (*6) layer, the compressive stress increases and restricts the insertion and diffusion of Li, leading to the emergence of inactive Si in the core region. As shown in Fig. 7e right, the optimal Si–C mass ratio (*4, corresponding to a Si : C ratio of 1 : 0.977) is determined from the cross-point values of the two lower limits in compressive stress and tensile stress, where both the maximum Si utilization and maintenance of structural integrity are achieved. Guided by such rational C–Si design, the densely compacted particles displayed a high tap density of 1.0–1.1 g cm^−3^ and a low specific surface area of 1.56 cm^2^ g^−1^. With an electrode compaction density of 1.6–1.65 g cm^−3^ (650 mA h g^−1^), a pouch-cell prototype demonstrates superior cycle stability.

While modeling and simulations provide theoretical analysis of the stress evolution and distribution inside or between the porous ATPs, the actual working environments of active particles in real battery cells are far more complex. This represents a key limitation of theoretical modeling. For example, in theoretical simulations, ideally uniform diffusion of Li-ions through a highly ordered structure is often assumed, but in actual electrodes the lithiation process may occur with SOC homogeneity and anisotropic structural changes. The non-linear and somewhat ambiguous mechanical properties of various intermediate Li_*x*_Si alloys further complicate this issue. For instance, a few simulation studies assumed that both Si and carbon were ideal Hookean elastic bodies capable of elastic deformation,^121^ but yielding and creeping of Li_*x*_Si species were experimentally identified, and their impact on lithiation kinetics remains unclear.^122^ Moreover, the mechanical attributes such as Poisson's ratio and Young's modulus are highly dependent on the dimensional scale; for instance, the modulus at the nanoscale can be orders of magnitude higher than that at the macroscopic scale. In other words, the mechanical responses of nano-sized subjects, which are often adopted as FEM elements, differ enormously from those of the ones in the practical batteries.^95,99^ Therefore, beyond theoretical simulations, advanced characterization tools are highly useful means to facilitate the understanding and design of porous ATPs, which will be expounded in the next section.

### 4.2 Diagnostic tools for intraparticle pores

Porosity characterization remains a critical metric for ATP powders, where conventional fluid-based methods (*e.g.*, mercury intrusion porosimetry [MIP] and gas adsorption [N_2_/CO_2_]) can provide fundamental parameters like pore size distribution and porosity. However, these methods offer limited information on pore connectivity and electrolyte infiltration behaviors at multiple scales (1–100 nm) as well as monitoring their evolution during cycling, which are the key factors influencing electrode performance. Advanced characterization enables holistic optimization of ATPs by providing multi-scale insights into the pore structure, electrolyte interaction, and degradation mechanisms. Moreover, providing more accurate mechanical and chemical parameters can facilitate the modeling of Si-based particles. As shown in Table 3, small-angle X-ray scattering (SAXS) can quantify closed pores inaccessible to fluids *via* Porod analysis, while small-angle neutron scattering (SANS) with contrast variation can distinguish pore-filling mechanisms (*e.g.*, electrolyte infiltration). Focused ion beam-SEM (FIB-SEM) provides nanoscale imaging of intraparticle pores, while nuclear magnetic resonance (NMR) relaxometry measures electrolyte mobility and pore wettability.

**Table 3:** Characterization methods for pore analysis of particle materials

Methods	Mechanism	Function for pores	Resolution
Fluid-based tests (MIP and N_2_/CO_2_ adsorption)	Fluid intrusion/physisorption	Porosity, pore shape, pore size distribution, and SSA	0.5 nm to 500 µm (MIP)
			0.5–50 nm (gas adsorption)
SAXS	Electron density contrast between pores and bulk	Closed porosity, pore tortuosity, and surface roughness	1–100 nm
SANS	Neutron scattering length density difference between pores and bulk	Pore-filling conditions, including electrolyte infiltration and Li plating	1–300 nm
FIB-SEM	Ion milling exposing particle interior + electron imaging of pores	Nanoscale cross-sectional imaging of pore networks and allowing 3D reconstruction	5 nm to 50 µm
(s)TEM	Electron diffraction/phase contrast	Atomic scale with high resolution, along with EELS chemical mapping	0.1 nm
NMR	Spin-lattice relaxation of fluids contained in solid pores	Pore wettability and accessibility for electrolyte	10 nm to 1 µm

**Fig. 8:**
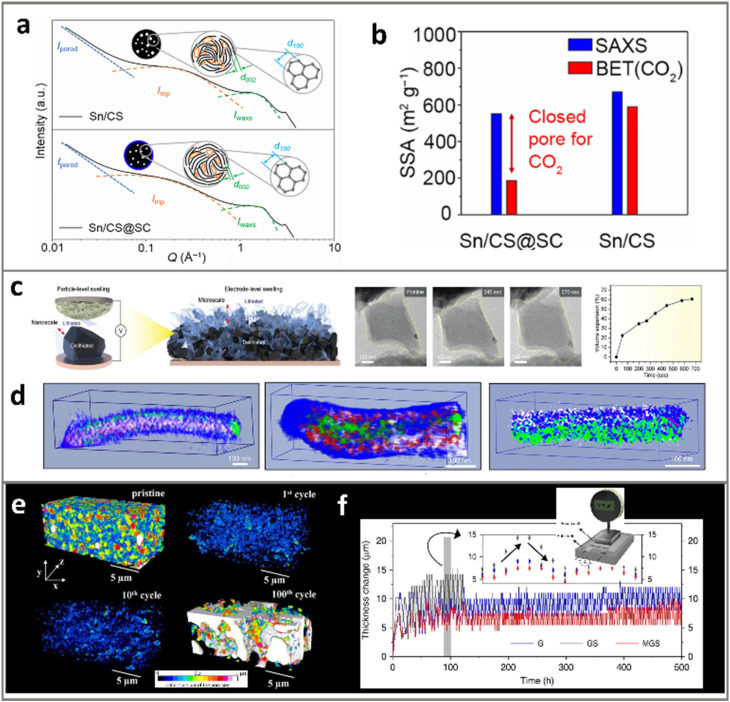
Advanced characterization studies for the pores in ATPs. The calculated method of closed-pores in Sn/CS@SC materials can be captured through combining full-range SAXS-WAXS analysis (a) and CO_2_ adsorption–desorption tests (b). Reprinted with permission from ref. 82. Copyright 2025 Elsevier. (c) *In situ* TEM captures the evolution of pores of graphene encapsulated Si during lithiation. Reprinted with permission from ref. 70. Copyright 2021 Wiley. (d) 3D cryo-STEM-EDS viewing of a Si nanowire at the 1st cycle, 36th cycle and 100th cycle, highlighting the growth of pores. Reprinted with permission from ref. 127. Copyright 2021 Springer (e) 3D FIB/SEM tomography identified the evolution of pore morphology and pore size distribution with respect to cycling. Reprinted with permission from ref. 135. Copyright 2016 IOP Publishing. (f) *In situ* pouch cell thickness measurement reflects varying electrode expansion during cycling. Reprinted with permission from ref. 110. Copyright 2019 Springer.

Jin *et al.*^82^ fabricated carbon spheres rich in sub-nanometer pores encapsulated with Sn single atoms (Sn/CS@SC) with sufficient closed pores. They first confirmed the existence of closed pores by the confinement and density calculation, due to the N_2_/CO_2_ inaccessibility of sub-nanopores. They further qualified the closed pores that are inaccessible to DMC through combined SAXS-WAXS techniques (Fig. 8a and b). Crucially, the persistence of SAXS peaks in Sn/CS@SC after cycling indicated effective electrolyte exclusion, while their disappearance in Sn/CS suggested pore filling by electrolyte and the SEI. This X-ray-based characterization, combined with varying powder-based density measurements, provides a comprehensive strategy for quantifying the closeness of pores. Such multimodal analysis is invaluable for designing porous ATPs as accessibility to electrolyte can dictate battery performance.

The evolution of pores in an ATP anode directly determines the electrochemical performances.^123–126^ Apart from pore characterization, it is of particular interest to probe and monitor chemical, mechanical, and geometrical evolution of the particles during cell operation. Shrinking of pores and sintering of nano-sized Li-alloys at high temperature and electrochemical cycling processes are known to be prevalent.^70^ Although using porous Si as a model simulation system greatly facilitates the scientific understanding, direct adoption of highly porous high-SSA (>10 m^2^ g^−1^) particles is impractical. Carbon–silicon composite particles represent a realistic venue for applying the porous ATPs for commercial practices.^115^ Due to the differing expansion ratio and Li-diffusivity of various constituents, *i.e.*, silicon, carbon, and pores, the evolution of phase boundaries and Li-diffusion paths in such composite secondary particles is highly intriguing but rarely explored.

Zhang *et al.*^70^ fabricated Si–carbon composite particles with a porosity of ∼20%, and *in situ* recorded their structural evolution *via* charged-coupled TEM (Fig. 8c). The particles were loaded on a gold wire probe as the working electrode, while Li-metal foil was scratched on a tungsten probe as the counter electrode, to constitute a half-cell miniature battery. During the lithiation, the shape extension of the particle was captured by TEM. Confirming the fully lithiated state by selected area electron diffraction, the volume change was precisely characterized: unlike the ∼300% expansion of bulk Si, the composite particle expands only 60.6% without fractures or cracks owing to the encapsulated pitch carbon and ample pores.

3D imaging techniques can visualize the complex intercorrelation among intraparticle components in the composite particles, with pore evolution being the primary focus in this case. To achieve *in situ* observation of the SEI and intraparticle pores, the cryogenic temperature can minimize the sample damage from air and the imaging e-beam. Wang *et al.*^127^ conducted cryo-STEM-EDS tomography on Si nanowires with diverse cycles to study the evolution of the 3D structure and elemental distribution (Fig. 8d). They demonstrated the occurrence of uneven SEI growth towards the Si interior during cycling. Evidence has shown that this inward SEI growth, being progressive and irreversible, is mediated by intraparticle pore formation due to Li vacancy condensation during delithiation. And these interconnected pores will further facilitate the permeation of the electrolyte molecules through the weakened and fractured SEI shell.

As advanced SEM and TEM offer insight into a single particle, the actual electrode performance is the collective result of numerous particles. Beyond the evolution inside the particle, *operando* monitoring of interparticle evolutions is equally critical. In practice, the particle-to-particle and particle-to-inactive-agent interactions also have prominent impacts on end-use performances.^128,129^ Earlier studies have shown that the most favored lithiation directions can be greatly altered by the imposed stress.^130^ Moreover, it is worth noting that most of the reported *in situ* TEM cells did not involve electrolytes, hence excluding the impact of the SEI, despite that the SEI often serves as the limiting factor of Li-ion diffusion and imposes a mechanical constraint.^131,132^ The action of stress at the particle surface can lead to great changes in bulk reactions, which single particle SEM and TEM may not capture.^133,134^

Roué *et al.*^135^ proposed FIB-SEM tomography that can monitor the pores at the electrode dimension. Electrode evolution at sub-50 nm resolution was reconstructed in a 20 × 8 × 11 µm^3^ field as shown in Fig. 8e. Drastic decreases in the porosity (from 57% at the 1st to 36% at the 100th cycle) were identified, suggesting that SEI debris filled the electrode pores. During the early stages, the pores shrink due to the filling of SEI products and enlarge in the late stage due to continuous particle expansion during cycling. This provides a detailed description of the evolution of electrode pores under varying cycling stages. Such electrode imaging and reconstruction can also be achieved in X-ray nano-tomography, where Chen-Wiegart *et al.*^136^ found that the electrode doubled in thickness and the nano-porous Si drifted away from the current collector upon the 2nd lithiation. After 500 cycles, the electrode is expanded 8 times, even much higher than the theoretical Si-to-Li_15_Si_4_ expansion. The cycle-induced development of macropores between the active particles and the delamination of particles from the current collector were reasons for such behavior.

Note that the stress or strain evolution at the electrode scale is rooted in not only the elastic or plastic deformation of active particles but also the external environment such as physical confinement, temperature, or static pressure. The real-time monitoring of the electrode in the cell scale is a key step in delving into this problem. Kim, Ko and Cho *et al.*^110^ employed a macropore-coordinated graphite–Si (MGS) hybrid, with Si being selectively deposited into the macropores. Such a composite material was adopted in a pouch-type full-cell, which was held under a thickness sensor while performing charge and discharge cycles to monitor the real-time thickness changes (Fig. 8f). The thickness fluctuated greatly in the early stage of cycling and continued to increase steadily in the later cycle period. This justifies the expansion of Si which could be effectively suppressed through the rational design of macropores. Well-regulated pore size is identified as key to avoid sacrificing particle compactness: tap density and SSA of MGS were at 1.13 g cm^−3^ and 2.21 m^2^ g^−1^, similar to graphite (1.08 g cm^−3^ and 3.13 m^2^ g^−1^). This coincides well with the previous discussion, where the crucial role of macropores in expansion-accommodation is emphasized.

In summary, as microscopy gives high spatial resolution down to the sub-nanoscale to reveal pore morphologies in targeted areas, incursion and spectroscopy-based tools are more powerful in providing quantitative and statistical analysis of the pores in bulk specimens. This includes pore size, pore volume, and pore closeness. With electrochemical *in situ* set-ups, real-time monitoring of the pore evolution enables direct correlation with electrochemistry. Building upon the advanced and ever-evolving analytical tools, it is now well understood that the pores determine many aspects of ATP performance, including the cycle, rate, and mechanical response. In this way, further understanding of the role of interparticle *versus* intraparticle pores and closed pores *versus* open pores was advocated.^4^

## 5. Electrode and electrolyte synergism for porous ATPs

As in practical electrodes, the ATP particles are processed and calendared to a thin and compact layer, and the electrochemical performance of electrodes is often heavily affected by the auxiliary components (conductive agents, binder, and current collector). In the following sections, we move to the discussion of electrode auxiliaries, focusing on the particle–auxiliary–component interactions and their effectiveness in stabilizing ATPs.

### 5.1 Binders and conductive agents for ATPs

Numerous studies reveal that Li–Si electrochemical alloying reactions can be stabilized through the use of novel binders, conductive agents (CAs), and electrolytes.^128,137–143^ A highly useful concept is the construction of a Si surface that can better bond with binders, be anchored with CAs, or form a stable SEI within electrolytes. For instance, a number of binder systems for Si-anodes were reported, such as carboxymethyl cellulose (CMC), alginate, poly(acrylic acid) (PAA), polyimide, and conductive polymers.^141,144,145^ As SiMPs are inherently denser and more compact than SiNPs, literature reported SiNPs often exhibit a TD of ∼0.2 g cm^−3^ and SSA of ∼60 m^2^ g^−1^,^84,146^ however, that of SiMPs are in the range of 0.4–1.0 g cm^−3^ and 5–50 m^2^ g^−1^.^125^ Therefore, in the following section, we adopt SiMPs as a model system to discuss their interactions with novel auxiliary components and the effectiveness in stabilizing Li–Si electrochemical alloying reactions.^142^

Yang and Wang *et al.*^147^ developed a self-healing binder, *i.e.*, poly(acrylic acid)-poly(2-hydroxyethyl acrylate-*co*-dopamine methacrylate, PAA-P(HEA-*co*-DMA)), for SiMPs. Compared with the traditional PAA binder, PAA-P(HEA-*co*-DMA) can withstand a very large strain. Moreover, its unique self-healing behavior can “heal” the cracks of SiMPs and prevent particle disintegration, enabling stable cycle performances at 3.2 mA h cm^−2^. Dai *et al.*^148^ proposed a 3D-conductive polymer binder by chemically bonding polyacrylic acid (PAA) onto amino-functionalized long single-wall carbon nanotubes (SCNTs). It is found that the immense mechanical degradation and pulverization of SiMPs could be alleviated by such a polymer binder. Under vacuum drying, the carboxyl groups (–COOH) of the PAA binder connect with the amino groups (–NH_2_) of SCNTs. Electrode peel forces increase to 81.85 N m^−1^ compared to that of CNT (24.59 N m^−1^) and carbon black (CB, 4.61 N m^−1^). As a result, the SiMP based electrode delivered a capacity of 3445.3 mA h g^−1^ with a high ICE of 89.7%. The electrodes stabilize at 10.59 mA h cm^−2^ with a Si loading of 5.37 mg cm^−2^.

Prior studies point out the critical role of the interaction between the binder and the surface of the ATPs. In that sense, the design of the binder should be system specific, *e.g.*, the differing surface characteristics of Si or C–Si or SiO_*x*_ particles would call for varying binder design.^149,150^ Song *et al.*^151^ proposed an interface-adaptive PSEA triblock polymer architecture for C–Si composite particles. The polymer chain is composed of three segments: (1) hydrophobic polystyrene, (2) elastic poly(2-(2-methoxyethoxy)ethyl acrylate), and (3) hydrophilic poly(acrylic acid). Such a supermolecular architecture was designed to form π⋯π stacking interactions with carbonaceous surfaces and hydrogen bonding with Si surfaces simultaneously. The supramolecular PSEA binder delivered higher adhesive strength (163 N m^−1^) compared to that of conventional PAA (24 N m^−1^) and NaCMC/SBR binders (100 N m^−1^). Benefiting from this design, cycling performances at practical areal capacities (4 mA h cm^−2^) were achieved.

The physical interactions between active materials and CAs also arouse great attention; one example is that the collapse of the conductive network will lead to capacity degradation.^152^ This case is extremely concerning for thick compact electrodes in practical cells, where particle-level volume change and dislocation are profound.^153,154^ Zhao, Yang, and Pan *et al.*^155^ designed a cross-linked conductive binder (CCB) for commercial micro-sized SiO_*x*_ anodes *via* covalently connecting a linear conductive binder (LCB) onto conjugated anchor points. The cross-linking structure of CCB dramatically improves not only the mechanical strength of the polymer but also its adhesive force as compared to the LCB. As a result, the SiO_*x*_/CCB electrode delivered a retention of 88.1% for 250 cycles at a high areal capacity of >2.1 mA h cm^−2^.

However, the over-dosage of auxiliary components in the electrode (inactive binder and CAs) is attracting increasing criticism. While commercial graphite electrodes have auxiliary components of less than 5 wt%, usually in the range of 2–5 wt%,^156,157^ the laboratory-grade electrode often adopts 10–20% or even more.^137,138,140,141,143,147,148,151,152,155,158–161^ Lowering the loading of auxiliary inactive components lies as a core demand in developing genuinely high-energy LIBs. Adopting conducting polymers to serve as a “one-for-all” multifunctional conductive binder has been explored.^138,140–142,144,145,151,162,163^ Due to the intrinsic differing chemical and physical properties of the polymeric binder and CAs (mostly carbons), especially the differing mechanical response to volume changes, cycle-induced degradation of physical contacts between the binder, CAs, and Si-containing active particles can be envisioned. Maintaining the binding force of the binder in dense, thick electrodes is even more arduous. Kim and Choi *et al.*^164^ proposed a capillary-inspired conductive agent (CCA) that possesses electron/ion dual-conductivity to serve as a multifunction binder. The CCA is composed of a polyanion and a CNT complex. Due to the superior conductivity of CNTs and electrolyte affinity of polyanions, a trace amount of CCA (∼2 wt%) enabled the graphite–silicon (GS) anode to perform stable cycles (85.4% after 180 cycles) at a high areal capacity of ∼3.5 mA h cm^−2^ and high TD of ∼1.5 g cm^−3^. Conversely, the GS electrode showed inferior cycle stabilities with conventional CA systems (∼75% for carbon black’ 82.7% for CNTs). Chen *et al.*^19^ developed a dual-conducting copolymer skin onto a Si surface, in which ionic conducting chains (PEG, polyethylene glycol) are crosslinked with the electrically conducting chains (PANi, polyaniline) at the macromolecular network. With robust covalent bonds, this dual-conducting coating realized superior Li-ion conductivity (>1.9 mS cm^−1^) and electronic conductivity (>0.4 S cm^−1^), and can protect the intraparticle hybrid alloying–plating reactions.

### 5.2 Advanced electrolytes for porous ATPs

Electrolyte is a key factor in stabilizing ATPs, by forming stable SEI layers and suppressing side reactions.^165–171^ Early studies point out the intrinsic instability of ATPs in conventional electrolyte systems. Typically, the electrolyte used in commercial LIB cells, *i.e.*, LiPF_6_ dissolved in mixed cyclic alkyl carbonate and linear carbonate solvents, presents poor compatibility with Si.^172^ For instance, fluoroethylene carbonate (FEC) and vinylene carbonate (VC) are found to suppress the reduction of other electrolyte components (*e.g.*, EC and DMC) by forming a LiF-rich or polymer-rich SEI that is thin, dense, and elastic.^173^ The unique SEI is thought to passivate the active particles and prevent further solvent permeation.^165,174–176^ Aside from FEC and VC, numerous electrolyte additives and electrolyte formulations emerged and were shown to stabilize ATPs. Huang and Zheng *et al.*^168^ utilized an ether-based electrolyte with FEC and lithium oxalyldifluoroborate (LiDFOB) dual additives (termed N-DHF), to stabilize nano-Si particles. With N-DHF, the formed SEI showed a high modulus that results in a high ICE of 90.2% and a low capacity-fading rate of 0.0615% per cycle (2041.9 mA h g^−1^ after 200 cycles) in the half cells. In the Si|NMC532 full cell (≈17.9 mg cm^−2^), N-DHF also enabled a capacity retention of 88.4% after 60 cycles. In contrast, the conventional F-control electrolyte only reached 20.3% retention at the same cycles.

**Fig. 9:**
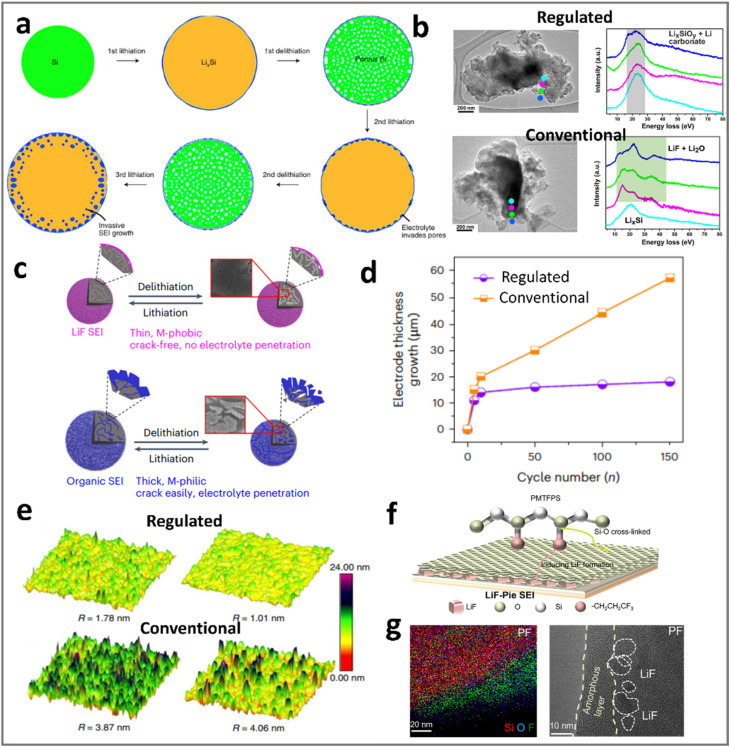
Advanced electrolytes for stabilizing porous ATPs. (a) Schematic of inward SEI growth of Si particles, due to cycle-induced transformation of Si into interconnected porous networks after cycles. Reprinted with permission from ref. 127. Copyright 2021 Springer. (b) The electron energy loss spectroscopy (EELS) imaging of the electrolyte decomposition products of cycled Si. (c) Schematics of the SEI differences in Si, where fluorinated electrolytes produce an ∼5 nm thin crack-free SEI while conventional electrolytes yield an ∼20 nm thick SEI, allowing electrolyte penetration. (d) The electrode thickness growth in varying electrolytes. Reprinted with permission from ref. 181. Copyright 2024 Springer. (e) The AFM surface roughness in conventional or regulated electrolyte at lithiated (left) and de-lithiated (right) states. Reprinted with permission from ref. 178. Copyright 2020 Springer. (f) Schematic of decomposition pathways of poly(methyl trifluoropropyl siloxane) (PMTFPS) and its interaction with the Si anode; (g) cryo-electron microscopy (cryo-EM) images of the SEI with a LiF-rich inner layer and cross-linked silane outerlayer. Reprinted with permission from ref. 185. Copyright 2025 Springer.

However, the by-product of F-rich electrolyte (*e.g.*, HF)^177^ and pulverization of particles^64^ will continuously demolish the interfacial stability. Wang *et al.*^127^ demonstrated that initially dense Si particles will evolve into a porous structure during cycling in a fluorinated carbonate electrolyte (1.2 M LiPF_6_ in EC : DMC = 3 : 7 wt% + 10 wt% FEC). As illustrated in Fig. 9a, they identified pore nucleation and growth as a consequence of the vacancy condensation due to Li-extraction during the first delithiation. The pores proximal to the particle surface consist of interconnected channels to allow the liquid electrolyte to penetrate and form the SEI layer on the freshly exposed inner surfaces. During repeated cycling, new pores progressively nucleate near existing pores, expanding and further extending the penetrating channels for electrolyte toward the particle interior, being an autocatalytic process.

There are many emerging studies on novel electrolyte systems that show the capability to prevent particle disintegration and inward pore propagation.^171^ Fluorinated ethers are drawing widespread attention due to the greatly improved compatibility with the lithium metal anode.^178–180^ In improving the reversibility of Li metal anodes, the formation of a dense F-rich SEI is thought to be the key, a concept analogous to enabling Si-anodes. Wang *et al.*^181^ regulated an asymmetric electrolyte by the introduction of solvent-free ionic liquids into a molecular solvent, which can conduct preferential anion reduction on the ATP (including Si, Al, Sn, and Bi) surface to form a dense LiF SEI. As shown in Fig. 9b, EELS analysis revealed that in this electrolyte, an ultrathin (3 nm), homogeneous LiF-rich SEI was formed where the LiF signal was sustained through the surface to the inner layer on micro-sized Si anodes. This is never achievable in conventional electrolyte (1.0 M LiPF_6_ in EC/DMC = 50/50 (wt/wt)). They further highlighted that the LiF-rich SEI has higher interfacial energy (*E*_int_) and weaker bonding to Li-alloying phases, key to ensuring the SEI shell integrity when the inner ATPs experience large volume changes. Within conventional electrolytes, the strong bonding between the organic SEI and lithiated ATP phases makes the SEI vulnerable, along with the particle pulverization (Fig. 9c). Furthermore, electrode thickness measurements have shown that in differing electrolytes, the cycle-induced electrode swelling can be suppressed with such unique SEI functionality (Fig. 9d).

The use of fluorinated ethers in combination with F-containing Li-salts has also been explored to stabilize ATP anodes.^170,171,182,183^ Lan and Zheng *et al.*^182^ formulated a localized high-concentration electrolyte (termed FEMC-LHCE), where 2,2,2-trifluoroethyl methyl carbonate (FEMC) is a key component to build a highly robust and stable F-rich inorganic–organic bilayer SEI on micron-sized Si particles. The fluorine moiety of FSI^−^ anions promoted the formation of a rigid inorganic inner SEI, while the FEMC solvent with the CF3-moiety contributed to an F-rich organic SEI. Stable cycling of SiMPs (62% retention after 150 cycles at 0.2C) was achieved at high areal capacity (3.4 mA h cm^−2^). The high anodic stability of the FEMC-LHCE was also proved by the stable operation of high-voltage SiMP|NMC811 Ah-level pouch-cells. Similarly, Zheng and Yamada *et al.*^184^ designed a cyclic phosphate (TFEP)/hydrofluoroether (HFE)-based electrolyte. A highly elastic and robust composite SEI mainly consisting of LiF, Li_2_O, Li_*x*_PO_*y*_, sulfur compounds, and polyphosphoesters, was identified on SiO microparticles. TFEP can form polymeric components, while HFE can intensify the Li^+^–FSI^−^ association to provide more FSI^−^ anions to participate in forming a thin and inorganic-rich SEI layer. Using 0.93 M LiFSI in TFEP/FEMC/HFE, a SiO|NCM622 full cell (∼590 Wh kg^−1^, based on the active masses) was fabricated to retain 71.4% capacity and 99.9% coulombic efficiency over 300 cycles.

For porous ATPs, where stress distribution is inherently more complex due to the tortuous pore structure, a uniform and high-modulus SEI can help mitigate localized stress concentrations and prevent pore collapse. Preventing the swelling, mud-cracking, or even exfoliation at the practical electrode level during repeated cycling can be highly relevant to battery performance. Wang *et al.*^178^ previously confirmed the surface morphology evolution of the LiF-based SEI *via in situ* electrochemical atomic force microscopy (AFM). As shown in Fig. 9e, the LiF–organic bilayer SEI formed by a regulated electrolyte (2.0 M LiPF6 in tetrahydrofuran/2-methyltetrahydrofuran (THF/MTHF)) effectively suppressed swelling of the Si electrode (∼1.78 nm during lithiation and ∼1.01 nm after delithiation). Such an SEI exhibits a superior uniform surface compared to the organic-dominated SEIs derived from conventional carbonate electrolytes (3.87 and 4.06 nm). This insight further points out the critical role of electrolytes in regulating SEIs, which has a profound impact on electrode swelling and interparticle pore evolution. Another electrolyte additive, poly(methyl trifluoropropyl siloxane) (PMTFPS), was introduced by Wang *et al.*^185^ to stabilize Si–C composite particles (Si nanograins embedded into porous carbon). Analogs to the “Apple Pie”, this SEI features a LiF-rich inner layer that enhances thermodynamic stability and mechanical rigidity, while a cross-linked silane outer matrix serves as the “crust” to accommodate expansion (Fig. 9f and e). The PMTFPS-based electrolyte reduces parasitic reactions, and hence achieves a capacity retention of 88.9% after 300 cycles of LiCoO_2_‖Si full cells, whereas in a conventional electrolyte it retained only 49.6%.

While stabilizing ATPs in liquid electrolyte remains challenging,^186,187^ solid-state electrolytes (SSEs) have recently emerged as promising candidates to pair with ATPs due to their nonvolatility and nonflowability.^188,189^ With the volume changes of ATPs being the well-known limiting factor in liquid electrolyte, the introduction of SSEs further imposes great challenges in controlling the formation and propagation of pores at the ATPs/SSEs interface. Cell performances will be greatly deteriorated where the insufficient electrolyte–electrode contact at interfaces increases to dictate the electrochemical reactions. The rigid solid–solid contact between brittle SSEs (*e.g.*, Li_7_La_3_Zr_2_O_12_) and ATPs exacerbates the strain mismatch, leading to interfacial detachment, pore formation, and crack propagation.^190^ Janek *et al.*^191^ studied the chemo-mechanical failure mechanisms at the solid–electrolyte–silicon interface. They detected the growth of interphases at the Si|Li_6_PS_5_Cl interface *via* time-of-flight secondary ion mass spectrometry (ToF-SIMS). The cycled Si|LPSCl interfaces showed that the decomposition products from LPSCl were responsible for the resistance increase. More importantly, the microscale pore formation at Si|LPSCl interfaces during de-lithiation predominantly aggravated the cell failure.

It is a consensus that the major task concentrates on improving the interfacial contact and suppressing pore propagation at the ATPs|SSEs interface. Under unconstrained free-surface conditions, *i.e.*, no external pressure applied to the cell stacks, the lithiation-induced expansion and interfacial failure escalate substantially. This can greatly deteriorate the lithiation kinetics and reversibility. Applying an appropriate external pressure is key to providing intimate interfacial contact and constraining porosity growth, collectively mitigating cell expansion and sustaining cycle life. Considering that SSEs and lithiated ATPs can be rather plastic, precise modulation of von Mises stress to a nuanced state should be advocated, where irreversible pore formation can be suppressed but without incurring engineering barriers of implementation (as in >10 MPa).

## 6. Future prospects

**Fig. 10:**
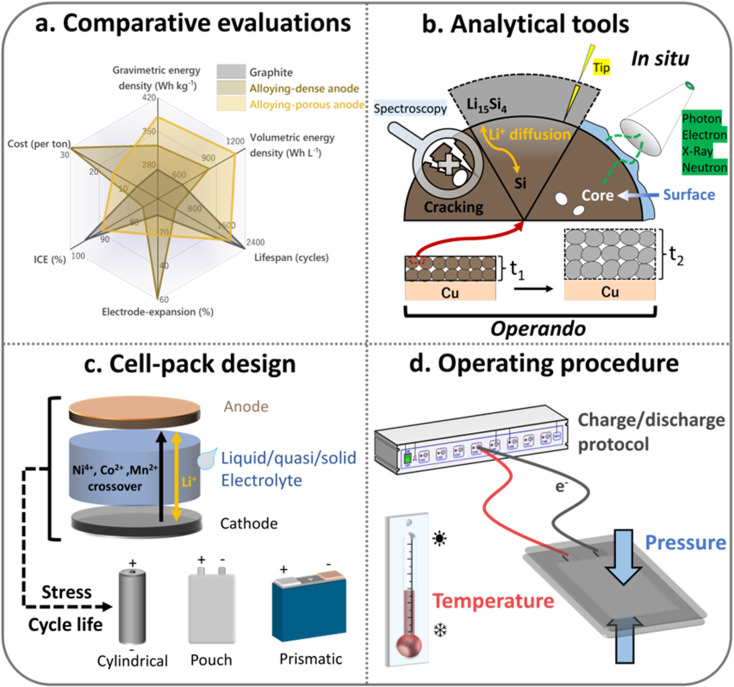
Research needs for further developments of porous ATP anodes. (a) End-use oriented evaluation of varying types of anodes using commercial graphite as the benchmark; (b) analytical tools to capture the dynamic evolution of intra- and inter-particle pores; (c) advanced cell-to-pack design that allows better monitoring and control of stress and strain levels for ATP based cells; (d) advanced operation procedures that integrate charge/discharge protocols and the associated temperature and pressure control.

The increasing adoption of alloying-type particles (ATPs) in the anode represents a major trend in advancing next-generation LIBs. Serving as a fulcrum in balancing the seemingly conflicting requirements of volume change and cell energy density, particle/electrode pore-engineering holds great promise. Despite the significant progress, several scientific and technological aspects require more effort to facilitate the deployment of porous ATPs. As “porous but dense” represents perhaps the most ideal but realistic concept in guiding future development, here we attempt to provide a holistic analysis of incentivizing further endeavors in transforming lab-scale advancements to applied technology in the LIB industry. These aspects are schematized in Fig. 10 and elaborated below.

### 6.1 Electrode evaluation benchmarks

Commercial graphite anode materials represent a formidable counterpart for offering comprehensive competitiveness in specific capacity (especially in volumetric metrics), cycle life, rate performances, and volume changes. Therefore, rigorously comparing the synthesized porous ATPs with prevailing commercial graphite under practical standards is necessary for all research endeavors. It should be noted that micro-sized particles^192^ and thick electrodes^24^ represent prevalent desires in next-generation LIBs; hence granulation and densification of ATPs are required. As shown in the radar charts in Fig. 10a, though dense ATP anodes showed superiority in many aspects to conventional graphite, their practical application remains heavily constrained by (1) electrode expansion, (2) cycle life span, and (3) prohibitive costs associated with nano-synthesis.

Pores engineered ATPs offer strong practical values, although achieving homogeneous pore distribution and optimal pore arrangement still requires much more intensive work. Despite the synthetic difficulties in safety and cost, CVD-depositing Si into the porous carbon host has already entered commercial practice and is expected to find niche applications in consumer electronics.^193,194^ Hence, we highlight that achieving ideal porous ATPs relies on closed-pore design, where the volume changes of the Li-alloying reaction can be accommodated without excessive electrolyte consumption and hindered Li desolvation. What is the ideal size, volume, and geometry of pores? How do the ATPs and the intraparticle pores evolve as Li comes in and out? These questions are rather complex and intermixed. Authentic advancements of practical porous ATPs rely on solving these questions and should be built upon the comparative evaluation against the commercial graphite benchmark.

### 6.2 Analytical tools

To date, an in-depth understanding of the interactions between the intraparticle pores and their surrounding substances, *i.e.*, active species, electrode auxiliaries, and electrolytes, is still lacking. Advanced analytical tools play a pivotal role in advancing these aspects, including synchronic-radiation and real-time *operando* characterization.^195–198^ These techniques, although frequently used in the LIB studies, are rarely explored in this regard. Real-time and high-resolution monitoring of intraparticle pores of ATPs, by means of scattering and microscopy, can greatly promote the understanding of its functions. This includes pore evolution at varying electrochemical states and their interaction with binders, conductive agents, and electrolytes. Moreover, while *in situ* analysis provides information on individual particles, the better approach would be tracking the particle under practical working conditions of LIBs, preferably with high spatial/time resolutions. More often, these approaches were referred to as “*operando* analysis”, which includes the real-time monitoring of the electrode thickness and microstructural changes in particles and pores in the electrode.

### 6.3 Cell-to-pack designs

The adoption of ATP-based materials has a significant impact on the safety and lifespan of cells, depending on the varying types of battery formats (cylindrical, pouch, and prismatic) as well as the module or pack configuration. This is especially relevant as electrode design with high loading (>3 mA h cm^−2^) and high compaction density (>1.6 g cm^−3^) becomes prevalent in commercial practice. One specific observation in the LIB industry is that when adopting Si-containing anodes, cylindrical/prismatic cells always perform better than pouch cells. One plausible explanation regards the rigid casing and physical confinement in helping maintain electrode structural integrity to survive large volume changes. We highlight that the expansion of electrodes need to be strictly controlled within a proper range to avoid separator failure and potential safety issues, being <30% of the double-sided negative electrode and <10–20% for multilayered jellyroll stack.^14,17,25^ Exploring the suitability of varying porous ATPs and the associated failure mechanism in differing cell formats, as well as re-designing porous ATPs for specific formats, are invaluable. As advanced binders, novel electrolytes (both liquid or solid) or current collectors have been proposed for upgrading ATPs, and a comprehensive and rigorous evaluation of these electrode auxiliaries in the context of practical full cells or modules is a critical step to push up the TRL (technology readiness level). We argue that only through practical cells using commercial graphite as a benchmarking reference can genuine advancement be made.

### 6.4 Cell operating procedures

The last but not least factor that may disrupt the field is battery cell operating procedures. There is ongoing debate that the best practices for testing the performance of LIBs targeting EV applications should shift to power-controlled charging and discharging protocols rather than conventional constant current/voltage, since the former is more relevant to automotive application scenarios with practical driving behaviors.^199^ In the context of porous ATPs, this may also directly relate to the service life and safety performance of LIBs. During which charging stages do the pores accommodate expansion most effectively? How to mitigate expansion through optimizing the charge–discharge procedures? Cell operation protocols for practical ATP anodes may include cut-off voltage, charge rates/power, SOCs, temperature, and external pressures.^200,201^ It has recently been identified that a short-voltage-pulse protocol can reactivate the isolated Si-particles within electrodes, achieving considerable capacity recovery.^202^ More effectively, the static pressure imposed onto the pouch cells dramatically impacts battery performances, in both solid and liquid electrolytes.^203,204^ We note that the pore evolution patterns and mechanical–electrochemical correlations of ATPs under varying static pressure levels are highly practical and yet to be established.

## 7. Summary

The review provides a holistic overview of alloying-type particles (ATPs, *e.g.*, Si, Sn, and Ge) with intraparticle pores for lithium-ion battery anodes. The available synthetic approaches of fabricating porous ATPs are introduced and analyzed in great detail in a case-by-case manner, including bottom-up assembly, top-down etching, and transcription deposition. Among all techniques, a pivotal concept is that the stable operation of ATPs in practical high-loading high-density LIBs relies on the manipulation of intraparticle pores. We also discussed recent progress in monitoring the in-cell evolution of pores and in a thorough understanding of intraparticle pores (open pores *versus* closed pores). We introduced advanced diagnostic tools for intraparticle pore characterization and novel binders/electrolytes for stabilizing porous ATPs, therefore addressing the hanging critical questions. How does the pore size impact its accessibility to varying types of electrolytes? How do the porosity and pore volume of the ATPs in their as-prepared state determine their end-use properties in practical LIBs, and why? Can we develop a synthetic technique capable of fine-tuning intraparticle pores that is both scalable and cost-effective in terms of $ per Ah? Addressing these questions is worth not only rigorous research but also the endeavours of the battery industry. In sum, this review goes beyond the synthetic chemistry of porous ATPs; it provides a prospect on how to build negative electrodes for next-generation LIBs.

## Author contributions

Yiteng Luo: conceptualization, investigation, writing – original draft. Sai Ho Pun: investigation. He Yan: supervision, writing – review & editing.

## Conflicts of interest

There are no conflicts to declare.

## Data Availability

No primary research results, software or code have been included and no new data were generated or analysed as part of this review.

## References

[cit1] Lübke E., Helfen L., Cook P., Mirolo M., Vinci V., Korjus O., Fuchsbichler B., Koller S., Brunner R., Drnec J., Lyonnard S. (2024). Energy Environ. Sci..

[cit2] Chen Z., Soltani A., Chen Y., Zhang Q., Davoodi A., Hosseinpour S., Peukert W., Liu W. (2022). Adv. Energy Mater..

[cit3] Obrovac M. N., Chevrier V. L. (2014). Chem. Rev..

[cit4] Luo Y. T., Chen Y. G., Koratkar N., Liu W. (2024). Adv. Sci..

[cit5] Agyeman D. A., Song K., Lee G.-H., Park M., Kang Y.-M. (2016). Adv. Energy Mater..

[cit6] Sun Y., Liu N., Cui Y. (2016). Nat. Energy.

[cit7] Xu Z. L., Liu X. M., Luo Y. S., Zhou L. M., Kim J. K. (2017). Prog. Mater. Sci..

[cit8] Li H., Li H., Lai Y., Yang Z., Yang Q., Liu Y., Zheng Z., Liu Y., Sun Y., Zhong B., Wu Z., Guo X. (2022). Adv. Energy Mater..

[cit9] Zhang C., Wang F., Han J., Bai S., Tan J., Liu J., Li F. (2021). Small Struct..

[cit10] Li P., Kim H., Myung S.-T., Sun Y.-K. (2021). Energy Storage Mater..

[cit11] Sun L., Liu Y., Shao R., Wu J., Jiang R., Jin Z. (2022). Energy Storage Mater..

[cit12] Huo H., Janek J. (2022). ACS Energy Lett..

[cit13] Zhang Y., Wu B., Mu G., Ma C., Mu D., Wu F. (2022). J. Energy Chem..

[cit14] Chae S., Choi S. H., Kim N., Sung J., Cho J. (2020). Angew Chem. Int. Ed. Engl..

[cit15] Chae S., Ko M., Kim K., Ahn K., Cho J. (2017). Joule.

[cit16] He S., Huang S., Wang S., Mizota I., Liu X., Hou X. (2020). Energy Fuels.

[cit17] Luo F., Liu B., Zheng J., Chu G., Zhong K., Li H., Huang X., Chen L. (2015). J. Electrochem. Soc..

[cit18] Ko M., Chae S., Ma J., Kim N., Lee H.-W., Cui Y., Cho J. (2016). Nat. Energy.

[cit19] Chen Z., Luo Y., Yang D., Hu Y., Hou H., Koratkar N., Zhou G., Liu W. (2025). Mater. Today.

[cit20] Cheng Z., Lin H., Liu Y., Yan Q., Su B.-L., Zhang H. (2025). Adv. Funct. Mater..

[cit21] Elango R., Demortière A., Andrade V., Morcrette M., Seznec V. (2018). Adv. Energy Mater..

[cit22] Zhou C.-C., Su Z., Gao X.-L., Cao R., Yang S.-C., Liu X.-H. (2021). Rare Met..

[cit23] Wu X., Xia S., Huang Y., Hu X., Yuan B., Chen S., Yu Y., Liu W. (2019). Adv. Funct. Mater..

[cit24] Kuang Y., Chen C., Kirsch D., Hu L. (2019). Adv. Energy Mater..

[cit25] Zhang C., Wang F., Han J., Bai S., Tan J., Liu J., Li F. (2021). Small Struc..

[cit26] Jia P., Guo J., Li Q., Liu Y., Zheng Y., Guo Y., Huang Y., Shen Y., Long L., Zhang H., Chen R., Zhang C., Zhang Z., Shen J., Dong S., Jiang J., Chang M., Liu X., Wang X., Tang Y., Shao H. (2025). Energy Environ. Sci..

[cit27] Sun L., Liu Y., Wang L., Jin Z. (2024). Adv. Funct. Mater..

[cit28] Imtiaz S., Amiinu I. S., Xu Y., Kennedy T., Blackman C., Ryan K. M. (2021). Mater. Today.

[cit29] Cheng Z., Jiang H., Zhang X., Cheng F., Wu M., Zhang H. (2023). Adv. Func. Mater..

[cit30] Yoshino A. (2012). Angew Chem. Int. Ed. Engl..

[cit31] Obrovac M. N., Christensen L. (2004). Electrochem. Solid-State Lett..

[cit32] Liu X., Wu X.-Y., Chang B., Wang K.-X. (2020). Energy Storage Mater..

[cit33] Ying H., Han W. Q. (2017). Adv. Sci..

[cit34] Wang M., Zhang F., Lee C. S., Tang Y. (2017). Adv. Energy Mater..

[cit35] Li Q. F., Bjerrum N. J. (2002). J. Power Sources.

[cit36] Obrovac M. N., Christensen L., Le D. B., Dahn J. R. (2007). J. Electrochem. Soc..

[cit37] Besenhard J., Hess M., Komenda P. (1990). Solid State Ionics.

[cit38] He J., Deng Y., Han J., Xu T., Qi J., Li J., Zhang Y., Zhao Z., Li Q., Xiao J., Zhang J., Kong D., Wei W., Wu S., Yang Q.-H. (2025). Nat. Commun..

[cit39] Lee H. J., Moon J. S., Byeon Y. W., Yoon W. Y., Kim H. K., Ahn J. P. (2022). ACS Energy Lett..

[cit40] Xu D. X., Zhao Y. M., Chen H. X., Lu Z. Y., Tian Y. F., Xin S., Li G., Guo Y. G. (2024). Angew Chem. Int. Ed. Engl..

[cit41] Song J., Chen S., Zhou M., Xu T., Lv D., Gordin M. L., Long T., Melnyk M., Wang D. (2014). J. Mater. Chem. A.

[cit42] Xu Z.-L., Gang Y., Garakani M. A., Abouali S., Huang J.-Q., Kim J.-K. (2016). J. Mater. Chem. A.

[cit43] Wang H., Fan S., Cao Y., Yang H., Ai X., Zhong F. (2020). ACS Appl. Mater. Interfaces.

[cit44] Kumar P., Berhaut C. L., Zapata Dominguez D., De Vito E., Tardif S., Pouget S., Lyonnard S., Jouneau P. H. (2020). Small.

[cit45] Guo J., Pei S., He Z., Huang L.-a., Lu T., Gong J., Shao H., Wang J. (2020). Electrochim. Acta.

[cit46] Lv Y., Shang M., Chen X., Nezhad P. S., Niu J. (2019). ACS Nano.

[cit47] Gan C., Zhang C., Wen W., Liu Y., Chen J., Xie Q., Luo X. (2019). ACS Appl. Mater. Interfaces.

[cit48] An Y., Tian Y., Wei H., Xi B., Xiong S., Feng J., Qian Y. (2019). Adv. Funct. Mater..

[cit49] Chen Y., Yuan Y., Xu C., Bao L., Yang T., Du N., Lin Y., Zhang H. (2020). J. Mater. Sci..

[cit50] Luo W., Chen X., Xia Y., Chen M., Wang L., Wang Q., Li W., Yang J. (2017). Adv. Energy Mater..

[cit51] Cheng Z., Jiang H., Zhang X., Cheng F., Wu M., Zhang H. (2023). Adv. Funct. Mater..

[cit52] Chen X., Wang B., Ye Y., Liang J., Kong J. (2024). Energy Environ. Mater..

[cit53] Wang W., Liu W., Wang Y., Gu S., Zheng H. (2025). Chem. Eng. J..

[cit54] Khan M., Yan S., Ali M., Mahmood F., Zheng Y., Li G., Liu J., Song X., Wang Y. (2024). Nano-Micro Lett..

[cit55] El Omari G., El Kindoussy K., Aqil M., Dahbi M., Alami J., Makha M. (2024). Heliyon.

[cit56] Ahmed H., Simoes dos Reis G., Molaiyan P., Lähde A., Lassi U. (2025). Prog. Energy.

[cit57] Zhang T., Wang T., Zheng Y., Qian L., Liu X., Yan W., Zhang J. (2025). Adv. Energy Mater..

[cit58] Sung J., Kim N., Ma J., Lee J. H., Joo S. H., Lee T., Chae S., Yoon M., Lee Y., Hwang J., Kwak S. K., Cho J. (2021). Nat. Energy.

[cit59] Wu Q., Ji X., Yu P., Cao Y., Li Z., Yu J., Huang Y. (2025). Nat. Protoc..

[cit60] Li W., Xu Y., Wang G., Xu T., Si C. (2024). Adv. Energy Mater..

[cit61] Chen F., Han J., Kong D., Yuan Y., Xiao J., Wu S., Tang D. M., Deng Y., Lv W., Lu J., Kang F., Yang Q. H. (2021). Natl. Sci. Rev..

[cit62] Xu Q., Sun J.-K., Li J.-Y., Yin Y.-X., Guo Y.-G. (2018). Energy Storage Mater..

[cit63] Liu Z., Lu D., Wang W., Yue L., Zhu J., Zhao L., Zheng H., Wang J., Li Y. (2022). ACS Nano.

[cit64] Li H., Chen Z., Kang Z., Liu W., Chen Y. (2023). Energy Storage Mater..

[cit65] Zhao Z., Han J., Chen F., Xiao J., Zhao Y., Zhang Y., Kong D., Weng Z., Wu S., Yang Q. H. (2022). Adv. Energy Mater..

[cit66] Yang Z., Liu C., Du Y., Yang Y., Jin H., Liu X., Ding F., Bai L., Ouyang Y., Yuan F. (2022). Comp. Part B: Eng..

[cit67] An W., Gao B., Mei S., Xiang B., Fu J., Wang L., Zhang Q., Chu P. K., Huo K. (2019). Nat. Commun..

[cit68] Choi S.-H., Nam G., Chae S., Kim D., Kim N., Kim W. S., Ma J., Sung J., Han S. M., Ko M., Lee H.-W., Cho J. (2019). Adv. Energy Mater..

[cit69] Han Z., Maitarad P., Yodsin N., Zhao B., Ma H., Liu K., Hu Y., Jungsuttiwong S., Wang Y., Lu L., Shi L., Yuan S., Xia Y., Lv Y. (2025). Nano-Micro Lett..

[cit70] Chae S., Xu Y., Yi R., Lim H. S., Velickovic D., Li X., Li Q., Wang C., Zhang J. G. (2021). Adv. Mater..

[cit71] Yang W., Ying H., Zhang S., Guo R., Wang J., Han W.-Q. (2020). Electrochim. Acta.

[cit72] Jia H., Li X., Song J., Zhang X., Luo L., He Y., Li B., Cai Y., Hu S., Xiao X., Wang C., Rosso K. M., Yi R., Patel R., Zhang J. G. (2020). Nat. Commun..

[cit73] Müller J., Abdollahifar M., Vinograd A., Nöske M., Nowak C., Chang S.-J., Placke T., Haselrieder W., Winter M., Kwade A., Wu N.-L. (2021). Chem. Eng. J..

[cit74] Xiao Y., Yi S., Yan Z., Qiu X., Ning P., Yang D., Du N. (2024). Small.

[cit75] Son Y., Kim N., Lee T., Lee Y., Ma J., Chae S., Sung J., Cha H., Yoo Y., Cho J. (2020). Adv. Mater..

[cit76] Bitew Z., Tesemma M., Beyene Y., Amare M. (2022). Sustain. Energy Fuels.

[cit77] Okuyama K., Abdullah M., Wuled Lenggoro I., Iskandar F. (2006). Adv. Powder Technol..

[cit78] Vertruyen B., Eshraghi N., Piffet C., Bodart J., Mahmoud A., Boschini F. (2018). Materials.

[cit79] Nandiyanto A. B. D., Okuyama K. (2011). Adv. Powder Technol..

[cit80] Joshi Y., Zamani S., Klaassen C., Joo Y. L. (2021). Adv. Energy Sustain. Res..

[cit81] Yang H., Lin S., Cheng A., He F., Wang Z., Wu Y., Zhang Y., Liu X. (2023). Energies.

[cit82] Jin T., Zhang X. Y., Yuan S., Yu L. (2025). Sci. Adv..

[cit83] Cooper A. R., Eaton L. E. (1962). J. Am. Ceram. Soc..

[cit84] Zhu X., Liu B., Shao J., Zhang Q., Wan Y., Zhong C., Lu J. (2023). Adv. Funct. Mater..

[cit85] Li X., Yan C., Wang J., Graff A., Schweizer S. L., Sprafke A., Schmidt O. G., Wehrspohn R. B. (2015). Adv. Energy Mater..

[cit86] Wang K., Tan Y., Li P., Sun J. (2020). Electrochim. Acta.

[cit87] Xiao Z., Wu H., Quan L., Zeng F., Guo R., Ma Z., Chen X., Zhan J., Xu K., Xing L., Li W. (2025). Energy Environ. Sci..

[cit88] Křenek T., Bezdička P., Murafa N., Šubrt J., Pola J. (2009). Eur. J. Inorg. Chem..

[cit89] Zhang A., Ringwala D. A., Mircovich M. A., Roldan M. A., Kouvetakis J., Menéndez J. (2024). J. Vac. Sci. Technol., A.

[cit90] Bencheikh M., El Farh L. (2024). Int. J. Hydrogen Energy.

[cit91] Hu M., Wu H., Zhang G.-J. (2023). Chem. Phys. Lett..

[cit92] Ito T., Hashimoto K., Shirai H. (2003). Jpn. J. Appl. Phys..

[cit93] Zhang Z., Zhang M., Wang Y., Tan Q., Lv X., Zhong Z., Li H., Su F. (2013). Nanoscale.

[cit94] Luo Y., Chen Y., Koratkar N., Liu W. (2024). Adv. Sci..

[cit95] Pharr M., Zhao K., Wang X., Suo Z., Vlassak J. J. (2012). Nano Lett..

[cit96] Liu B., Jia Y., Li J., Jiang H., Yin S., Xu J. (2020). J. Power Sources.

[cit97] Duan S., Laptev A. M., Mücke R., Danilov D. L., Notten P. H. L., Guillon O. (2019). Mech. Res. Commun..

[cit98] Wang D., Wang Y., Zou Y., Lu C., Ma Z. (2018). Acta Mech..

[cit99] Zuo X., Wen Y., Qiu Y., Cheng Y. J., Yin S., Ji Q., You Z., Zhu J., Muller-Buschbaum P., Ma L., Bruce P. G., Xia Y. (2020). ACS Appl. Mater. Interfaces.

[cit100] Li X., Gu M., Hu S., Kennard R., Yan P., Chen X., Wang C., Sailor M. J., Zhang J. G., Liu J. (2014). Nat. Commun..

[cit101] Shenoy V. B., Johari P., Qi Y. (2010). J. Power Sources.

[cit102] Zhao K., Wang W. L., Gregoire J., Pharr M., Suo Z., Vlassak J. J., Kaxiras E. (2011). Nano Lett..

[cit103] Wang H., Chew H. B. (2016). Extreme Mech. Lett..

[cit104] Sethuraman V. A., Chon M. J., Shimshak M., Srinivasan V., Guduru P. R. (2010). J. Power Sources.

[cit105] Liu X. H., Zheng H., Zhong L., Huang S., Karki K., Zhang L. Q., Liu Y., Kushima A., Liang W. T., Wang J. W., Cho J.-H., Epstein E., Dayeh S. A., Picraux S. T., Zhu T., Li J., Sullivan J. P., Cumings J., Wang C., Mao S. X., Ye Z. Z., Zhang S., Huang J. Y. (2011). Nano Lett..

[cit106] McDowell M. T., Lee S. W., Nix W. D., Cui Y. (2013). Adv. Mater..

[cit107] Lee S. W., McDowell M. T., Choi J. W., Cui Y. (2011). Nano Lett..

[cit108] Yang H., Huang S., Huang X., Fan F., Liang W., Liu X. H., Chen L. Q., Huang J. Y., Li J., Zhu T., Zhang S. (2012). Nano Lett..

[cit109] Rohrer J., Albe K. (2013). J. Phys. Chem. C.

[cit110] Ma J., Sung J., Hong J., Chae S., Kim N., Choi S. H., Nam G., Son Y., Kim S. Y., Ko M., Cho J. (2019). Nat. Commun..

[cit111] Wang H., Lu S.-H., Wang X., Xia S., Beng Chew H. (2021). J. Phys. D: Appl. Phys..

[cit112] Zhang S. (2017). npj Comput. Mater..

[cit113] Ge M., Rong J., Fang X., Zhou C. (2012). Nano Lett..

[cit114] Srinivasan R., Chandran K. S. R. (2023). J. Power Sources.

[cit115] Han X., Zhang Z., Zheng G., You R., Wang J., Li C., Chen S., Yang Y. (2019). ACS Appl. Mater. Interfaces.

[cit116] Liu X. H., Huang J. Y. (2011). Energy Environ. Sci..

[cit117] Liu B., Xu J. (2020). ACS Appl. Energy Mater..

[cit118] Gao X., He P., Ren J., Xu J. (2019). Energy Storage Mater..

[cit119] Lee Y., Lee T., Hong J., Sung J., Kim N., Son Y., Ma J., Kim S. Y., Cho J. (2020). Adv. Funct. Mater..

[cit120] Qi C., Li S., Yang Z., Xiao Z., Zhao L., Yang F., Ning G., Ma X., Wang C., Xu J., Gao J. (2022). Carbon.

[cit121] Simolka M., Heim C., Friedrich K. A., Hiesgen R. (2019). J. Electrochem. Soc..

[cit122] Fan F., Huang S., Yang H., Raju M., Datta D., Shenoy V. B., van Duin A. C. T., Zhang S., Zhu T. (2013). Model. Simulat. Mater. Sci. Eng..

[cit123] Ko M., Chae S., Cho J. (2015). ChemElectroChem.

[cit124] Nzabahimana J., Liu Z., Guo S., Wang L., Hu X. (2020). ChemSusChem.

[cit125] Zhu G., Chao D., Xu W., Wu M., Zhang H. (2021). ACS Nano.

[cit126] Cheng Z., Jiang H., Zhang X., Cheng F., Wu M., Zhang H. (2023). Adv. Funct. Mater..

[cit127] He Y., Jiang L., Chen T., Xu Y., Jia H., Yi R., Xue D., Song M., Genc A., Bouchet-Marquis C., Pullan L., Tessner T., Yoo J., Li X., Zhang J.-G., Zhang S., Wang C. (2021). Nat. Nanotechnol..

[cit128] Bie Y., Yang J., Nuli Y., Wang J. (2017). J. Mater. Chem. A.

[cit129] Ling M., Xu Y., Zhao H., Gu X., Qiu J., Li S., Wu M., Song X., Yan C., Liu G., Zhang S. (2015). Nano Energy.

[cit130] Lee S. W., Lee H. W., Ryu I., Nix W. D., Gao H., Cui Y. (2015). Nat. Commun..

[cit131] Zhou X., Liu Y., Du C., Ren Y., Mu T., Zuo P., Yin G., Ma Y., Cheng X., Gao Y. (2018). J. Power Sources.

[cit132] Ko M., Chae S., Jeong S., Oh P., Cho J. (2014). ACS Nano.

[cit133] Galvez-Aranda D. E., Verma A., Hankins K., Seminario J. M., Mukherjee P. P., Balbuena P. B. (2019). J. Power Sources.

[cit134] Ren Y., Yin X., Xiao R., Mu T., Huo H., Zuo P., Ma Y., Cheng X., Gao Y., Yin G., Li Y., Du C. (2022). Chem. Eng. J..

[cit135] Etiemble A., Tranchot A., Douillard T., Idrissi H., Maire E., Roué L. (2016). J. Electrochem. Soc..

[cit136] Zhao C., Wada T., De Andrade V., Gürsoy D., Kato H., Chen-Wiegart Y.-c. K. (2018). Nano Energy.

[cit137] Li Z., Zhang Y., Liu T., Gao X., Li S., Ling M., Liang C., Zheng J., Lin Z. (2020). Adv. Energy Mater..

[cit138] Jiao X., Yin J., Xu X., Wang J., Liu Y., Xiong S., Zhang Q., Song J. (2020). Adv. Funct. Mater..

[cit139] Chen Z., Wang C., Lopez J., Lu Z., Cui Y., Bao Z. (2015). Adv. Energy Mater..

[cit140] Lee H. A., Shin M., Kim J., Choi J. W., Lee H. (2021). Adv. Mater..

[cit141] Chen H., Wu Z., Su Z., Chen S., Yan C., Al-Mamun M., Tang Y., Zhang S. (2021). Nano Energy.

[cit142] Deng L., Zheng Y., Zheng X., Or T., Ma Q., Qian L., Deng Y., Yu A., Li J., Chen Z. (2022). Adv. Energy Mater..

[cit143] Li S., Liu Y.-M., Zhang Y.-C., Song Y., Wang G.-K., Liu Y.-X., Wu Z.-G., Zhong B.-H., Zhong Y.-J., Guo X.-D. (2021). J. Power Sources.

[cit144] Zhao H., Wei Y., Qiao R., Zhu C., Zheng Z., Ling M., Jia Z., Bai Y., Fu Y., Lei J., Song X., Battaglia V. S., Yang W., Messersmith P. B., Liu G. (2015). Nano Lett..

[cit145] Choi S., Kwon T. W., Coskun A., Choi J. W. (2017). Science.

[cit146] Shi J., Gao H., Hu G., Zhang Q. (2022). Mater. Today Energy.

[cit147] Xu Z., Yang J., Zhang T., Nuli Y., Wang J., Hirano S.-i. (2018). Joule.

[cit148] Zhang B., Liu D., Xie H., Wang D., Hu C., Dai L. (2022). J. Power Sources.

[cit149] Shi Q., Zhou J., Ullah S., Yang X., Tokarska K., Trzebicka B., Ta H. Q., Rümmeli M. H. (2021). Energy Storage Mater..

[cit150] Zuo X., Zhu J., Müller-Buschbaum P., Cheng Y.-J. (2017). Nano Energy.

[cit151] Hu L., Jin M., Zhang Z., Chen H., Boorboor Ajdari F., Song J. (2022). Adv. Funct. Mater..

[cit152] Song Z., Zhang T., Wang L., Zhao Y., Li Z., Zhang M., Wang K., Xue S., Fang J., Ji Y., Pan F., Yang L. (2022). Small Methods.

[cit153] Li L., Zuo Z., Pan H., Chang Q., Gao X., Zhai X., Li Y. (2022). J. Power Sources.

[cit154] Tan W., Wang L., Lu Z., Yang F., Xu Z. (2022). Small.

[cit155] Song Z., Chen S., Zhao Y., Xue S., Qian G., Fang J., Zhang T., Long C., Yang L., Pan F. (2021). Small.

[cit156] Hawley W. B., Li J. (2019). J. Energy Storage.

[cit157] Kwade A., Haselrieder W., Leithoff R., Modlinger A., Dietrich F., Droeder K. (2018). Nat. Energy.

[cit158] Bridel J. S., Azaïs T., Morcrette M., Tarascon J. M., Larcher D. (2009). Chem. Mater..

[cit159] Beattie S. D., Larcher D., Morcrette M., Simon B., Tarascon J. M. (2008). J. Electrochem. Soc..

[cit160] Dong P., Zhang X., Zamora J., McCloy J., Song M.-K. (2023). J. Energy Chem..

[cit161] Choi S., Kwon T.-w., Coskun A., Choi J. W. (2017). Science.

[cit162] Higgins T. M., Park S. H., King P. J., Zhang C. J., McEvoy N., Berner N. C., Daly D., Shmeliov A., Khan U., Duesberg G., Nicolosi V., Coleman J. N. (2016). ACS Nano.

[cit163] Kim J., Kim G., Park Y. K., Lim G., Kim S. T., Jung I. H., Kim H. (2023). Adv. Funct. Mater..

[cit164] Kwon J., Kim J., Bae S. Y., Jeon S. P., Song J. H., Wang S. E., Jung D. S., Jang J., Park H., Kim P. J., Choi J. (2022). J. Power Sources.

[cit165] Yang Y., Li Z., Xu Y., Yang Z., Zhang Y., Wang J., Xu H., He X., Zhao H. (2023). J. Power Sources.

[cit166] Wang J., Cui Y. (2020). Nat. Energy.

[cit167] Wölke C., Sadeghi B. A., Eshetu G. G., Figgemeier E., Winter M., Cekic-Laskovic I. (2022). Adv. Mater. Interfaces.

[cit168] Cao Z., Zheng X., Qu Q., Huang Y., Zheng H. (2021). Adv. Mater..

[cit169] Johnson N. M., Yang Z., Kim M., Yoo D.-J., Liu Q., Zhang Z. (2022). ACS Energy Lett..

[cit170] Cao Z., Zheng X., Zhou M., Zhao T., Lv L., Li Y., Wang Z., Luo W., Zheng H. (2022). ACS Energy Lett..

[cit171] Chae S., Kwak W.-J., Han K. S., Li S., Engelhard M. H., Hu J., Wang C., Li X., Zhang J.-G. (2021). ACS Energy Lett..

[cit172] Xu K. (2014). Chem. Rev..

[cit173] Li J. L., Wang Y. N., Sun S. Y., Zheng Z., Gao Y., Shi P., Zhao Y. J., Li X., Li Q., Zhang X. Q., Huang J. Q. (2024). Adv. Energy Mater..

[cit174] Markevich E., Salitra G., Aurbach D. (2017). ACS Energy Lett..

[cit175] Hou T., Yang G., Rajput N. N., Self J., Park S.-W., Nanda J., Persson K. A. (2019). Nano Energy.

[cit176] Xu C., Lindgren F., Philippe B., Gorgoi M., Björefors F., Edström K., Gustafsson T. (2015). Chem. Mater..

[cit177] Kim N., Kim Y., Sung J., Cho J. (2023). Nat. Energy.

[cit178] Chen J., Fan X., Li Q., Yang H., Khoshi M. R., Xu Y., Hwang S., Chen L., Ji X., Yang C., He H., Wang C., Garfunkel E., Su D., Borodin O., Wang C. (2020). Nat. Energy.

[cit179] Cheng X. B., Zhang R., Zhao C. Z., Zhang Q. (2017). Chem. Rev..

[cit180] Kwon H., Baek J., Kim H.-T. (2023). Energy Storage Mater..

[cit181] Li A.-M., Wang Z., Lee T., Zhang N., Li T., Zhang W., Jayawardana C., Yeddala M., Lucht B. L., Wang C. (2024). Nat. Energy.

[cit182] Liu Y., Huang Y., Xu X., Liu Y., Yang J., Lai J., Shi J., Wang S., Fan W., Cai Y. P., Lan Y. Q., Zheng Q. (2023). Adv. Funct. Mater..

[cit183] Jia H., Zou L., Gao P., Cao X., Zhao W., He Y., Engelhard M. H., Burton S. D., Wang H., Ren X., Li Q., Yi R., Zhang X., Wang C., Xu Z., Li X., Zhang J. G., Xu W. (2019). Adv. Energy Mater..

[cit184] Yang S., Zhang Y., Li Z., Takenaka N., Liu Y., Zou H., Chen W., Du M., Hong X.-J., Shang R., Nakamura E., Cai Y.-P., Lan Y.-Q., Zheng Q., Yamada Y., Yamada A. (2021). ACS Energy Lett..

[cit185] Li W., Xu S., Zhong C., Fang Q., Weng S., Ma Y., Wang B., Li Y., Wang Z., Wang X. (2025). Nano-Micro Lett..

[cit186] Xiao Z., Lin X., Zhang C., Shen J., Zhang R., He Z., Lin Z., Jiang H., Wei F. (2023). Small Methods.

[cit187] Lee C. R., Jang H. Y., Leem H. J., Lee M. A., Kim W., Kim J., Song J. H., Yu J., Mun J., Back S., Kim H. s. (2023). Adv. Energy Mater..

[cit188] Xiao Z., Zou Z., Zhao K., Lin Z., Zhang B., Yu Y., Zhu C., Xu K., Xing L., Li W. (2025). Adv. Mater..

[cit189] Chen Y., Qian J., Wang K., Li L., Wu F., Chen R. (2025). Adv. Mater..

[cit190] Zhao N., Khokhar W., Bi Z., Shi C., Guo X., Fan L.-Z., Nan C.-W. (2019). Joule.

[cit191] Huo H., Jiang M., Bai Y., Ahmed S., Volz K., Hartmann H., Henss A., Singh C. V., Raabe D., Janek J. (2024). Nat. Mater..

[cit192] Zhao Z., Chen F., Han J., Kong D., Pan S., Xiao J., Wu S., Yang Q. H. (2023). Adv. Energy Mater..

[cit193] Costantino H. , Sakshaug A., Dhanabalan A. and Timmons C., Low Cost Manufacturing of Advanced Silicon-Based Anode Materials, 2019

[cit194] Rathore D., Abraham J. J., Mendel-Elias E., Li Z., Zaker N., Amirkhiz B. S., Johnson M., Hamam I., Leontowich A. F. G., Bond T., Dahn J. R. (2025). J. Electrochem. Soc..

[cit195] Cao S., Zhu Z., Zhang W., Xia H., Zeng Y., Yuan S., Ge X., Lv Z., Wei J., Liu L., Du Y., Xi S., Loh X. J., Chen X. (2023). Adv. Mater..

[cit196] Sun G., Yu F. D., Lu M., Zhu Q., Jiang Y., Mao Y., McLeod J. A., Maley J., Wang J., Zhou J., Wang Z. (2022). Nat. Commun..

[cit197] Yuan X., Liu B., Mecklenburg M., Li Y. (2023). Nature.

[cit198] Zeng D., Yao J., Zhang L., Xu R., Wang S., Yan X., Yu C., Wang L. (2022). Nat. Commun..

[cit199] Hatzell K., Chang W., Bao W., Cai M., Glossmann T., Kalnaus S., Liaw B., Meng Y. S., Mohtadi R., Wang Y. (2024). Joule.

[cit200] Lee D.-C., Lee K.-J., Kim C.-W. (2019). International Journal of Precision Engineering and Manufacturing-Green Technology.

[cit201] Quinn J. B., Waldmann T., Richter K., Kasper M., Wohlfahrt-Mehrens M. (2018). J. Electrochem. Soc..

[cit202] Yang Y., Biswas S., Xu R., Xiao X., Xu X., Zhang P., Gong H., Zheng X., Peng Y., Li J., Ai H., Wu Y., Ye Y., Gao X., Serrao C., Zhang W., Sayavong P., Huang Z., Chen Z., Cui Y., Vila R. A., Boyle D. T., Cui Y. (2024). Science.

[cit203] Liu W., Luo Y., Hu Y., Chen Z., Wang Q., Chen Y., Iqbal N., Mitlin D. (2024). Adv. Energy Mater..

[cit204] Tan D. H. S., Chen Y.-T., Yang H., Bao W., Sreenarayanan B., Doux J.-M., Li W., Lu B., Ham S.-Y., Sayahpour B., Scharf J., Wu E. A., Deysher G., Han H. E., Hah H. J., Jeong H., Lee J. B., Chen Z., Meng Y. S. (2021). Science.

